# TSPAN6 is a suppressor of Ras-driven cancer

**DOI:** 10.1038/s41388-022-02223-y

**Published:** 2022-02-19

**Authors:** Patrick O. Humbert, Tamara Zoranovic Pryjda, Blanka Pranjic, Andrew Farrell, Kohei Fujikura, Ricardo de Matos Simoes, Rezaul Karim, Ivona Kozieradzki, Shane J. F. Cronin, G. Gregory Neely, Thomas F. Meyer, Astrid Hagelkruys, Helena E. Richardson, Josef M. Penninger

**Affiliations:** 1 Department of Biochemistry & Genetics, School of Molecular Sciences, La Trobe University, Bundoora, VIC 3086 Australia; 2 Institute of Molecular Biotechnology of the Austrian Academy of Science, Dr. Bohrgasse 3, 1030 Vienna, Austria; 3 Max Planck Institute for Infection Biology, Charite Platz 1, 10117 Berlin, Germany; 4 Department of Medical Genetics, Life Sciences Institute, University of British Columbia, Vancouver, BC Canada; 5 Center for Integrative Bioinformatics Vienna, Max F. Perusetz Laboratories, Dr.-Bohr-Gasse 9/6, A-1030 Vienna, Austria; 6 Dr. John and Anne Chong Lab for Functional Genomics, Charles Perkins Centre, School of Life and Environmental Sciences, The University of Sydney, Sydney, NSW 2006 Australia; 7 Laboratory of Infection Oncology, Institute of Clinical Molecular Biology, Christian Albrecht’s University of Kiel and University Hospital Schleswig Holstein—Campus Kiel, Kiel, Germany

**Keywords:** Cell polarity, Growth factor signalling, Cancer genetics

## Abstract

Oncogenic mutations in the small GTPase RAS contribute to ~30% of human cancers. In a *Drosophila* genetic screen, we identified novel and evolutionary conserved cancer genes that affect Ras-driven tumorigenesis and metastasis in *Drosophila* including confirmation of the tetraspanin Tsp29Fb. However, it was not known whether the mammalian Tsp29Fb orthologue, TSPAN6, has any role in RAS-driven human epithelial tumors. Here we show that TSPAN6 suppressed tumor growth and metastatic dissemination of human *RAS* activating mutant pancreatic cancer xenografts. Whole-body knockout as well as tumor cell autonomous inactivation using floxed alleles of *Tspan6* in mice enhanced *Kras*^*G12D*^-driven lung tumor initiation and malignant progression. Mechanistically, TSPAN6 binds to the EGFR and blocks EGFR-induced RAS activation. Moreover, we show that inactivation of *TSPAN6* induces an epithelial-to-mesenchymal transition and inhibits cell migration in vitro and in vivo. Finally, low *TSPAN6* expression correlates with poor prognosis of patients with lung and pancreatic cancers with mesenchymal morphology. Our results uncover TSPAN6 as a novel tumor suppressor receptor that controls epithelial cell identify and restrains RAS-driven epithelial cancer.

## Introduction

The 3 RAS proteins (H-RAS, N-RAS, and K-RAS) are small GTPase binary molecular switches, that switch between active (GTP bound) and inactive (GDP bound) states [1]. Members of the RAS family are central players in many signaling networks and are amongst the most commonly identified oncogenes in human cancer [2, 3]. Gain of function mutations of *RAS*, such as *RASV12*, that increase the levels of the GTP-bound version of the protein were first observed in cancers approximately 40 years ago [4]. Activating mutation in *RAS* leads to a cascade of molecular events affecting cell proliferation, cell survival, and, thus, lead to carcinogenesis [5, 6]. Although activating *RAS* mutations are common, this event is rarely sufficient to promote tumorigenesis, as it promotes cellular senescence [7–10]. As such, additional mutations or silencing of tumor suppressors is frequently needed to promote tumorigenesis and metastasis together with activation of the RAS signaling pathway [11–13].

In order to identify novel tumor suppressors that cooperate with activated *Ras* (*RasV12*) to promote tumorigenesis, we undertook a genome-wide genetic screen using transgenic RNAi lines in the vinegar fly, *Drosophila melanogaster*, model organism [14]. This screen identified many novel potential tumor suppressor genes, and we verified that 80 out of the top 100 genes whose orthologs were downregulated in human cancer were able to cooperate with RasV12 to promote epithelial tissue overgrowth in *Drosophila*. One of these conserved tumor suppressor genes was *Drosophila Tsp29Fb*, the mammalian ortholog of which is *TSPAN6* (*TM4SF6*) [14]. Tetraspanins are small transmembrane proteins expressed in all multicellular organisms, which have been implicated in multiple biological functions [15]. They are known to interact with one another, other transmembrane molecules such as integrins and other adhesion receptors as well as growth factor and cytokine receptors to modulate cellular signaling [16, 17]. Tetraspanin mammalian family members, such as CD9, CD63, CD81, CD82, CD151, or Net2, have previously been implicated in tumorigenesis [18, 19]. In contrast to many human Tetraspanin family members with broad expression [20–22], TSPAN6 mRNA and protein are primarily expressed in epithelial cells. Further to this, we found that the *Drosophila TSPAN6* ortholog, *Tsp29Fb*, regulates epithelial architecture and EGFR-Ras signaling [14]. Intriguingly, Tsp29Fb was also found to affect the junctional localization of the Dlg1 cell polarity protein and genetically interact with the Scribble (Scrib)/Dlg1 polarity module [14]. In *Drosophila*, loss of function mutations of the cell polarity genes *dlg1* and *scrib* lead to uncontrolled proliferation, loss of epithelial polarity and invasive phenotypes, and they cooperate with oncogenic *Ras* in tumorigenesis in both *Drosophila* and mouse models [23–27].

As the function of TSPAN6 in mammalian systems was unknown, in this current study we investigate the role of TSPAN6 in human cell lines and mouse cancer models. We show that the *TSPAN6* knockdown mirrors the loss of either human *SCRIB* or *DLG1* in the presence of *H-RASV12*, leading to an increase in invasive spiking in MCF10A normal human breast cells grown in 3D conditions and in 2D cultures *TSPAN6* knockdown reduces epithelial sheet migration, similar to *SCRIB* and *DLG1* knockdowns. Furthermore, we show that human TSPAN6 acts as a tumor suppressor in a mouse orthotopic model of pancreatic cancer, and that full body as well as tissue specific knockout of *Tspan6* in mice enhances lung cancer driven by activated *Kras*. Mechanistically, TSPAN6 binds to the EGFR and suppresses EGFR-RAS-ERK signaling and promotes an epithelial cell morphology in vitro and in vivo. Furthermore, we show in lung and pancreatic cancer cohorts that low *TSPAN6* expression correlates with poor survival in cancers with mesenchymal features. Altogether, our results reveal TSPAN6 as a novel tumor suppressor gene that cooperates with activated RAS.

## Results

### Human *TSPAN6* knockdown phenocopies *SCRIB* and *DLG1* knockdowns in affecting cell migration and invasive phenotypes in vitro

**Fig. 1:**
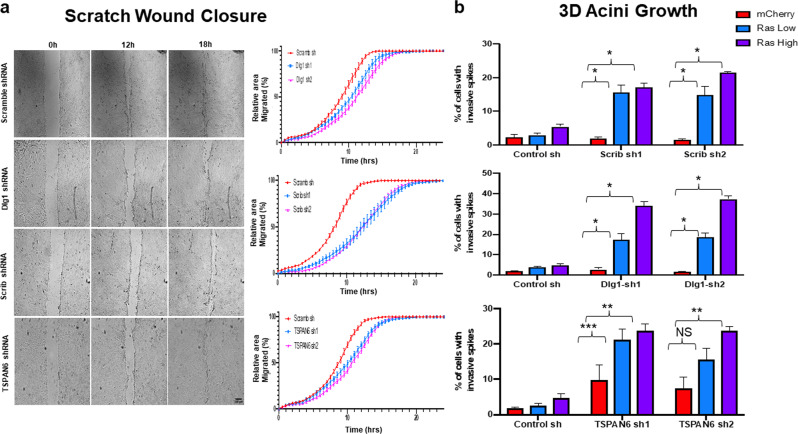
TSPAN6 controls epithelial cell migration and cooperates with H-RASV12 to induce cell invasion in normal human mammary epithelial cells. **a** The knockdown of *SCRIB*, *DLG1*, and *TSPAN6* leads to decreased epithelial sheet migrative potential. Representative phase contrast microscope images of *Scrib*, *Dlg1*, or *TSPAN6* knockdown MCF10A epithelial cell monolayers at 0, 12, and 18 h after wounding, and quantification of wound closure from 0–24 h. In each case, the corresponding shRNA constructs had a slower wound closing time as compared to the *Scramble* shRNA control (*SCRIB* sh vs *Scram* *P* < 0.0001; *DLG1* sh vs *Scram* *P* < 0.0001; *TSPAN6* sh vs *Scram* *P* < 0.0001; two-way ANOVA, 3 independent experiments). Scale bar = 100 μM. **b** *SCRIB*, *DLG1* and *TSPAN6* knockdown promote *H-RASV12*-induced invasion in 3D cultures. In combination with low or high levels of *H-RASV12*, knockdown of *SCRIB*, *DLG1*, and *TSPAN6* led to increased invasive acini in 3D culture. (*P < 0.0001, ** *P* < 0.01, ***P < 0.05; one-way ANOVA, three independent experiments). See Supplementary data for microscope images.

Given our findings in *Drosophila* that *Tsp29Fb* genetically interacts with *scrib* [14], we first sought to test whether TSPAN6 functions similarly to Scrib and Dlg1 in mammalian cells. In mammalian systems, both Dlg1 and Scrib have also been implicated in wound healing and the coordinated movement of epithelial cells [28, 29] (Galea and Humbert, unpublished data), and *SRIB* or *DLG1* knockdown cooperates with *H-RASV12* to drive cell invasion in 3D epithelial cell cultures [11, 24, 30] (Galea and Humbert, unpublished data). To determine whether human TSPAN6 also regulates cell migration and invasion similarly to SCRIB and DLG1, we generated MCF10A breast epithelial cell lines where *SCRIB*, *DLG1* or *TSPAN6* were stably knocked down (using 2 independent hairpins) in the presence of absence of oncogenic *H-RASV12*. 2D culture scratch wound assays, which measure epithelial sheet migration, a directed cell migration, revealed that knockdown of *TSPAN6* phenocopied the knockdown of *SCRIB* and *DLG1* in inhibiting wound closure, with a delay of ~6 h relative to the control (Fig. 1a). This result indicates that, similar to the function of SCRIB and DLG1, epithelial sheet migration is positively regulated by TSPAN6.

We then examined the cooperation of *TSPAN6* knockdown with *H-RASV12* in 3D MCF10A cultures, where *SCRIB* or *DLG1* knockdown have been shown to cooperate with *H-RASV12* in inducing an invasive phenotype [11]. Knockdown of *TSPAN6*, *SCRIB*, or *DLG1* alone did not alter acini morphology, although occasional invasive spikes were observed in *TSPAN6* knockdown cells (Fig. 1b, Supplementary Fig. 1). Whereas low or high expression of *H-RASV12* in cells resulted in a small number of invasive spikes in the acini, we found that knockdown of *TSPAN6*, greatly increased the number of invasive spikes in acini, similar to the knockdown of *SCRIB* or *DLG1* (Fig. 1b and Supplementary Fig. 1) [11]. Thus, TSPAN6 acts similarly to SCRIB and DLG1 in preventing cell invasion in *H*-*RASV12*-transformed epithelial cells.

### Tspan6 controls growth and metastases of Ras-transformed epithelial cells in vivo

**Fig. 2:**
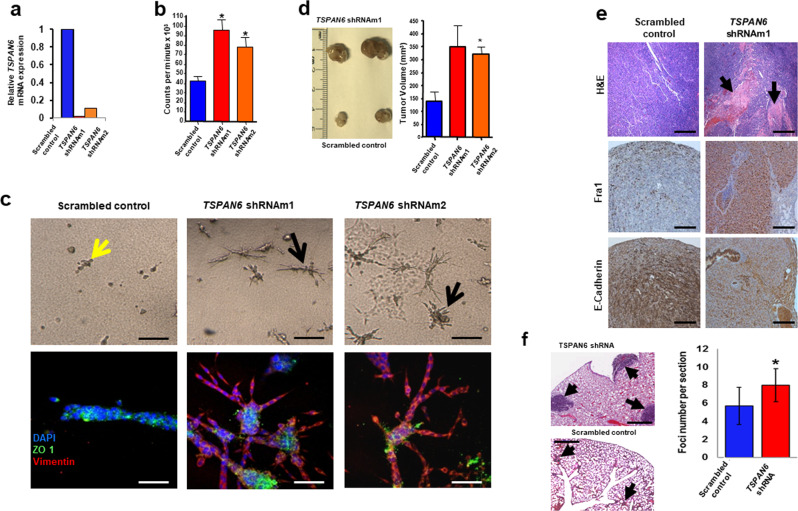
*Tspan6* knockdown in EpRas mouse mammary epithelial cells affects cell polarity, proliferation, and invasion. **a** Efficacy of shRNA mediated *Tspan6* knockdown in murine mammary epithelial EpRas cells. Data for two different shRNA knockdown lines, termed shRNAm1 and shRNAm2, are shown relative to the *scrambled* shRNA control. Cells were transfected using a lentiviral approach. Data were obtained by qPCR. **b** Proliferation of EpRas mammary cancer cells infected with lentiviral vectors encoding two distinct shRNAs targeting *Tspan6*, or *scrambled* shRNA as a control. Proliferation was determined in triplicate at 8 h using tritiated thymidine incorporation (mean ± SEM). Experiments were conducted twice. **c** Knockdown of *Tspan6* results in EMT in *H-Ras*^*V12*^ transformed epithelial EpRas cells. Top panels show representative cell morphologies of EpRas cells transfected with *scrambled* shRNA (control) or two distinct shRNAs targeting *Tspan6* 5 days after seeding of 5 × 10^3^ cells in collagen gels. Experiments were conducted twice. Yellow arrow indicates typical tubular structures. Black arrows indicate spindle-like cell morphologies indicative of EMT. Lower panel shows immunostaining for the epithelial marker ZO-1 (green) and the mesenchymal marker vimentin (red). DAPI (blue) is shown to label nuclei. Images were taken by confocal microscopy. Scale bars, 80μM top panels, 50μM bottom panels. **d** Left: Representative macroscopic appearances of tumors from control EpRas and *Tspan6* knockdown EpRas cells 4 weeks after inoculation. EpRas cells transfected with lentiviruses targeting *Tspan6* or *scrambled* shRNA (control) were inoculated in mammary fat pads of female *nu/nu* hosts (2.5 × 10^5^ cells each). Experiments were conducted twice. Right: Mean tumor volume 4 weeks after inoculation (n = 8 per group). *P < 0.05 (Student’s *t* test). **e** In vivo tumor growth, and effect on Fra1 and E-cadherin expression. Top panel, Hematoxylin and Eosin (H&E) staining of cross tumor sections from designated xenografts. Black arrows indicate large necrotic areas formed by EpRas *Tspan6* shRNA cells. Middle panel, Fra1 immunostaining in tumors from control EpRas and *Tspan6* knockdown EpRas cells showing that the Ras pathway target, Fra1 is upregulated in *Tspan6* knockdown tumors. Bottom panel, E-cadherin immunostaining in tumors from control EpRas and *Tspan6* knockdown EpRas cells indicate dissolution of adherens junction upon *Tspan6* knockdown. Representative images are shown at 4 weeks after injection into female *nu/nu* hosts. Scale bars = 1000 μM. **f** Lung metastases of control (scrambled shRNA) and *Tspan6* knockdown murine EpRas epithelial cells 30 days after tail vein injection of 5 × 10^5^ cells/mouse. Arrows indicate metastases. Scale bar is 1000 µm. For quantification (right panel), at least ten different planes from each lung were H&E stained and analyzed in a blinded fashion. Data are from at least 4 mice per genotype, and experiments were conducted twice. Mean foci numbers ± s.e.m. per lung section are shown. *p < 0.05 (Student’s *t* test).

To address the functional role of Tspan6 in Ras-mutant cancers, we first examined the effect of reduced *Tspan6* expression in mouse mammary epithelial cells (Eph4) stably transfected with oncogenic *H-Ras*, termed EpRas cells [31]. Stable knockdown of *Tspan6* in Ras-transformed EpRas cells (Fig. 2a) increased proliferation in vitro (Fig. 2b). EpRas cells exhibit a stable epithelial phenotype and normally undergo an epithelial-to-mesenchymal transition (EMT) only upon TGFβ treatment [31]. Intriguingly, *RNAi*-mediated knockdown of *Tspan6* in EpRas cells by itself was sufficient to induce an EMT-like phenotype, with cells invading the collagen 3D matrix showing reduction in ZO-1 and gain of Vimentin expression (Fig. 2c).

To study the in vivo tumorigenic potential, we performed orthotopic injection of EpRas ± *Tspan6-shRNA* cells into the mammary fat pad of female *nu/nu* mice. Stable knockdown of *Tspan6* in Ras-transformed EpRas cells indeed enhanced tumor growth in vivo following orthotopic injection (Fig. 2d). Immunohistochemistry of the in vivo tumors formed by EpRas cells with stable *Tspan6* knockdown revealed upregulation of the Ras pathway target, Fra1, and strongly reduced E-cadherin expression (Fig. 2e), indicative of an EMT [32]. Since we did not observe metastases in this orthotopic injection model, we performed tail vein injections and assayed for lung metastases. Stable depletion of *Tspan6* in EpRas cells significantly increased numbers and sizes of lung metastases as compared to cells transduced with *scrambled* control *RNAi* (Fig. 2f). Taken together, our experiments using stable knockdown of endogenous *Tspan6* in *H-Ras*-transformed mouse epithelial cells identify Tspan6 as a regulator of *RAS*-oncogene dependent epithelial identity, tumor growth, and invasion/metastasis.

### TSPAN6 controls growth and metastases of human *K-RAS* mutant pancreatic cancer by interacting with the EGFR

**Fig. 3:**
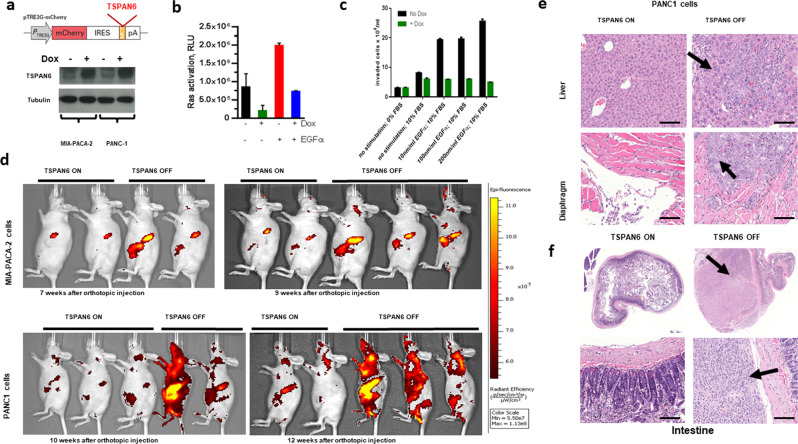
TSPAN6 inhibits growth and metastasis of activating *RAS* mutant human epithelial tumor cells. **a** Schematic vector map of doxycycline (Dox) inducible mCherry-IRES-TSPAN6. Bottom panel shows doxycycline-inducible TSPAN6 protein expression in stably transfected MIA PaCa2 and PANC1 cells. **b** Ras activation assay measured upon EGFα stimulation in PANC1 in the absence of or upon induced *TSPAN6* expression. Experiments were performed in six repeats. **c** Transwell migration of *TSPAN6*-PANC1 cells in response to treatment with EGFα and/or growing FBS gradient. For each condition, experiments were carried out in triplicate. **d** Representative bioluminescence images of *nu/nu* recipient mice orthotopically transplanted with the Dox-inducible *TSPAN6* human pancreatic cancer cell lines MIA PaCa2 (upper panels) and PANC1 (lower panels) in the presence or absence of Dox. **e** Representative H&E staining of liver and diaphragm sections of *TSPAN6* ON and *TSPAN6* OFF PANC1 cohorts 12 weeks after orthotopic grafting, showing reduction of metastatic foci (arrows) upon *TSPAN6* expression. Scale bars = 200 μM. **f** Representative intestinal sections (H&E staining) of mice following orthotopic injection of *TSPAN6* ON and *TSPAN6* OFF PANC1 cells. Scale bars = 500 μM.

Next, to determine the role of elevated expression of *TSPAN6* in suppressing *RAS*-mutant human cancers in vivo, we used a doxycyclin (Dox)-inducible Tet ON/OFF switch to regulate *TSPAN6* expression in two human *K-RAS* activating mutant pancreatic cancer cell lines, MIA PaCa2 and PANC1 (Fig. 3a and Supplementary Fig. 2a). We used these two pancreatic adenocarcinoma cell lines because they carry constitutively activated endogenous *K-RAS* mutations (*K-RASG12C* and *K-RASG12D* respectively) and in an initial pre-screen of a panel of pancreatic cancer cell lines, we observed low expression of *TSPAN6* in these lines compared to others examined (data not shown). Overexpression of *TSPAN6* in both cell lines reduced RAS activation at baseline and, importantly, following EGFR stimulation (Fig. 3b and Supplementary Fig. 2b). Using phospho-kinase arrays we observed that induction of *TSPAN6* led to reduced phosphorylation of EGFR, ERK1/2, and P38 at basal levels and following EGFR stimulation (Supplementary Fig. 2c). Interestingly, the Phospho-3-Inositol Kinase (PI3K) arm of EGFR-RAS signaling (indicated by pAKT1/2/3 and pTOR) was not similarly repressed upon *TSPAN6* expression in EGF-stimulated cells (Supplementary Fig. 2c), and in fact the phosphorylation of AKT1/2/3 and TOR was increased, suggesting that *TSPAN6* inhibits EGFR-RAS-ERK signaling, but has the opposite effect upon PI3K-AKT-TOR signaling. Reduced EGF-induced phosphorylation in *TSPAN6* expressing PANC1 cells was confirmed using an antibody that detects phosphorylated tyrosine 1173 of the activated EGFR (Supplementary Fig. 2d), indicating that TSPAN6 affects proximal EGFR signaling. Thus, similar to the function of *Drosophila* Tsp29Fb, mammalian TSPAN6 represses RAS activation and EGFR signaling.

Functionally, we found that Dox-induced *TSPAN6* expression in both MIA PaCa2 and PANC1 pancreas cancer cells decreased proliferation at baseline (Supplementary Fig. 2e). Moreover, *TSPAN6* overexpression reduced EGF-dependent invasion using transwell migration assays (Fig. 3c); in vitro scratch assays further confirmed that *TSPAN6* overexpression in these *K*-*RAS* activating mutant pancreatic cancer cells leads to impaired epithelial sheet migration (Supplementary Fig. 2f). EGFR stimulation had no apparent effect on the ability of TSPAN6 to inhibit proliferation (Supplementary Fig. 2e), but markedly increased invasion, which was abrogated by *TSPAN6* expression (Fig. 3c). Furthermore, Dox-induced *TSPAN6* expression in EGFα-stimulated PANC1 cells reduced the expression of the EMT markers, N-Cadherin and Vimentin (Supplementary Fig. 3a), and also reduced the expression of the EMT transcription factor, Slug, in both EGFα-stimulated PANC1 and MIA PaCa2 cells (Supplementary Fig. 3b), indicating that *TSPAN6* expression dictates an epithelial cell state.

Importantly, when we implanted control (no Dox) and Dox-induced MIA PaCa2 and PANC1 cells orthotopically into the pancreas of immunodeficient *nu/nu* mice, *TSPAN6* overexpression markedly reduced tumor growth within the primary pancreatic injection site and significantly decreased metastatic spread to distant sites (Fig. 3d–f and Supplementary Fig. 4a–e). Thus, overexpression of *TSPAN6* in the two *K-RAS*-activating mutant human pancreatic cancer cell lines, PANC1 and MIA PaCa2, impaired proliferation and migration in vitro and significantly reduced tumor growth and metastasis in vivo.

**Fig. 4:**
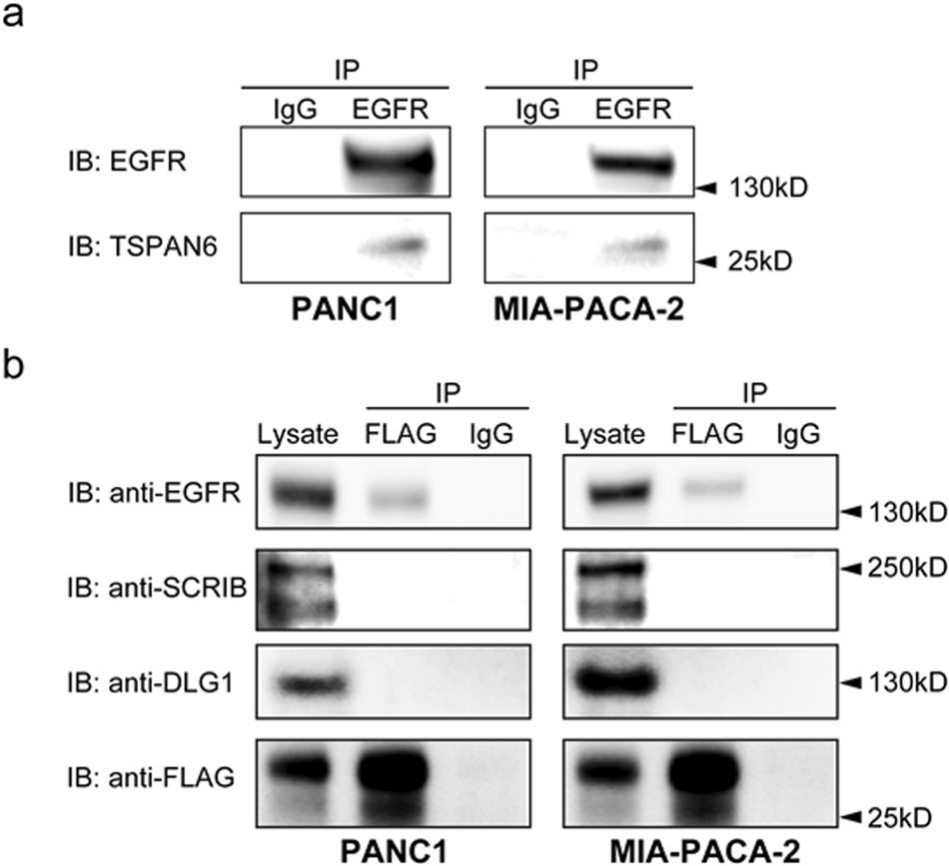
TSPAN6 Interacts with the EGFR but not with SCRIB or DLG1. **a** Co-immunoprecipitation between TSPAN6 and the EGFR. PANC1 or MIA-PaCa2 cells were lysed in 1% Brij 98 protein extraction buffer. After immunoprecipitation (IP) with anti-EGFR or IgG control antibodies, proteins were separated by SDS-PAGE and detected by immunoblotting with anti-TSPAN6 or anti-EGFR antibodies (IB). **b** Co-immunoprecipitation between FLAG-TSPAN6 overexpressing pancreatic cell lines and the EGFR. PANC1 or MIA-PaCa2 cells were lysed in 1% Brij 98 protein extraction buffer. After immunoprecipitation (IP) with anti-FLAG or IgG control antibodies, proteins were separated by SDS-PAGE and detected by immunoblotting with anti-EGFR, anti-SCRIB, anti-DLG1, or anti-FLAG antibodies (IB) The Lysate is also shown. Experiments were conducted once.

To further explore how Tspan6 was regulating EGFR-RAS signaling we examined whether TSPAN6 interacts with EGFR in the PANC1 and MIA PaCa2 cells. Endogenous TSPAN6 was co-immunoprecipitated with EGFR under mild stringency conditions (1% Brij 98) (Fig. 4a), but not under stringent conditions (0.5% Triton X100) (not shown). Additionally, using the converse IP approach, an interaction was also observed between TSPAN6 and EGFR when extracts from cells expressing a Flag tagged-TSPAN6 were immunoprecipitated with an anti-FLAG antibody (Fig. 4b). The interaction observed between TSPAN6 and EGFR only under mild stringency conditions is consistent with previous observations that tetraspanins form a multimolecular transmembrane complex called the “tetraspanins web” that is maintained in nonionic detergent [21, 33]. We also investigated the interaction between TSPAN6 and DLG1-SCRIB complex and did not observe any interactions (Fig. 4b). Altogether the results showing that TSPAN6 and the EGFR physically interact suggests that TSPAN6 directly regulates the EGFR. Moreover, since TSPAN6 does not interact with DLG1 or SCRIB it suggests that TSPAN6 and DLG1/SCRIB regulate EGFR-RAS signaling by parallel pathways.

### Tspan6 controls *Kras*^*G12D*^ driven lung cancer in vivo

**Fig. 5:**
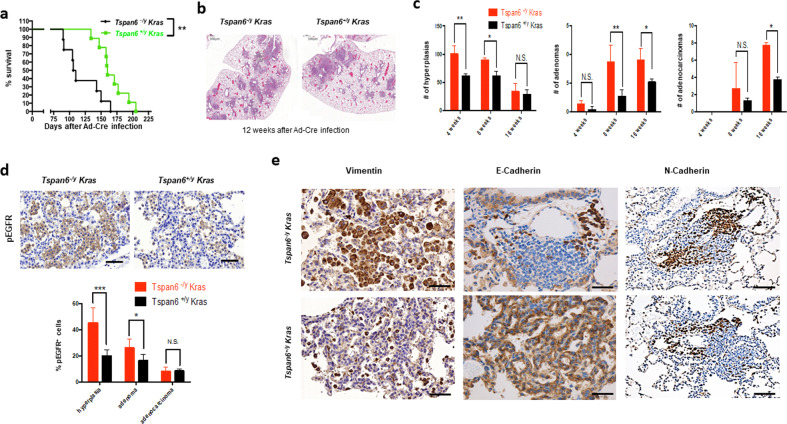
Tspan6 is a tumor suppressor in *KRas*^*G12D*^-driven lung cancer. **a** Kaplan–Meier survival plots for whole-body *Tspan6*^*-/y*^ *Kras*^*G12D*^ mice (*n* = 8) and *TSPAN6*^*+/y*^ *Kras*^*G12D*^ (*n* = 9) littermate controls. Log-rank test was used for statistical analysis. **P < 0.01. **b** Representative histological H&E sections of *Tspan6*^*-/y*^ *Kras*^*G12D*^ and *Tspan6*^*+/y*^ *Kras*^*G12D*^ lungs 12 weeks after Ad-Cre infection. Magnifications x5. **c** Quantification of hyperplasic regions, adenomas, and adenocarcinomas in *Tspan6*^*-/y*^ *Kras*^*G12D*^ and *Tspan6*^*+/y*^ *Kras*^*G12D*^ lungs at the indicated time points following Ad-Cre inhalation. *n* = 5 for each cohort and time point analyzed. Data are shown as means ± s.e.m. * *P* < 0.05; ** *P* < 0.01; N.S. = not significant (Student’s *t* test). **d** Representative in situ immunostaining for phosphorylated EGFR (pEGFR) 8 weeks post Ad-Cre delivery to lungs of *Tspan6*^*-/y*^ *Kras*^*G12D*^ (*n* = 5) and *Tspan6*^*+/y*^ *Kras*^*G12D*^ mice (*n* = 5). Right panel shows quantification of % pEGFR^+^ cells in hyperplasic regions, adenomas, and adenocarcinomas. Data are shown as mean ± s.e.m. * *P* < 0.05; ***P < 0.001; N.S. = not significant (Student’s *t* test). Scale bars = 200 μM. **e** Representative immunostaining for the mesenchymal markers, vimentin, and N-cadherin, and the epithelial marker E-cadherin in lung tumors 8 weeks after Ad-Cre inhalation. Sections were counterstained with haematoxylin. Scale bars, top left panel = 100 μM, bottom left panel = 200 μM, middle panels = 200 μM, right panels = 500 μM.

To obtain definitive proof that Tspan6 plays a role in Ras-driven tumorigenesis, we generated whole-body *Tspan6* mutant mice using gene targeting via homologous recombination (Supplementary Fig. 5a, b). Homozygous mutant mice were born at the expected Mendelian frequency and exhibited normal fertility with no obvious anatomical or histological abnormalities. *Tspan6* mutant mice were then crossed to mice carrying the *Lox-Stop-Lox-Kras*^*G12D*^ transgene. *Lox-Stop-Lox-Kras*^*G12D*^ mice develop non-small-cell lung carcinomas (NSCLCs) upon Cre-mediated deletion and clonal induction of the mutant *Kras*^*G12D*^ allele in a well-described stepwise process that leads from epithelial hyperplasia and benign adenomas to adenocarcinomas [34]. We used male *Tspan6* mutant offspring (*Tspan6*^*−/y*^ *Kras*^*G12D*^) and their *Tspan6* expressing littermates (*Tspan6*^*+/y*^ *Kras*^*G12D*^) as controls since *Tspan6* is located on the X-chromosome. Clonal activation of *Kras*^*G12D*^-expression by adeno-viral Cre delivery (Ad-Cre) [34] significantly reduced survival in the *Tspan6*-knockout background compared to their respective littermate controls (P < 0.01, Fig. 5a). Quantification of overall tumor burden revealed a significant increase of the tumor areas in the lungs of *Tspan6*^*−/y*^ *Kras*^*G12D*^ mice compared to *Tspan6*^*+/y*^ *Kras*^*G12D*^ littermates (Fig. 5b and Supplementary Fig. 5c). We next assessed tumor initiation and staged the malignant progression of the lung cancers. At 4 weeks after Ad-Cre inhalation, *Tspan6*^*−/y*^ *Kras*^*G12D*^ mice harbored significantly more hyperplastic lesions than their *Tspan6* expressing littermates (Fig. 5c). Eight weeks after Ad-Cre inhalation, we again observed significantly more hyperplastic regions as well as increased numbers of adenomas in *Tspan6*^*−/y*^ *Kras*^*G12D*^ mice (Fig. 5c). At 16 weeks after Ad-Cre infection, knockout of *Tspan6* resulted in markedly increased progression to adenocarcinomas (Fig. 5c). These data show that genetic inactivation of *Tspan6* results in enhanced *Kras*^*G12D*^-driven tumor initiation and malignant lung cancer progression.

Proliferation, as detected by Ki67 staining, was significantly enhanced in hyperplastic regions, as well as in early (4 weeks after Ad-Cre inhalation) adenomas and adenocarcinomas from *Tspan6*^*−/y*^ *Kras*^*G12D*^ mice compared to their *Tspan6*^*+/y*^ *Kras*^*G12D*^ littermates (Supplementary Fig. 5d, e). We detected only a few apoptotic cells in *Kras*^*G12D*^-induced lung tumors from both *Tspan6* mutant and wild-type mice and we did not find significant differences in the percentages of apoptotic cells among the different cohorts (not shown). Immunohistochemistry for phospho-EGFR revealed markedly increased phospho-EGFR staining in *Tspan6* mutant lung tumors, in particular in the hyperplastic regions and adenomas (Fig. 5d). We also detected enhanced expression of vimentin and N-cadherin, as well as reduced E-cadherin expression in the *Tspan6*^*−/y*^ *Kras*^*G12D*^ lung tumors (Fig. 5e), further supporting the notion that loss of Tspan6 induces an EMT.

**Fig. 6:**
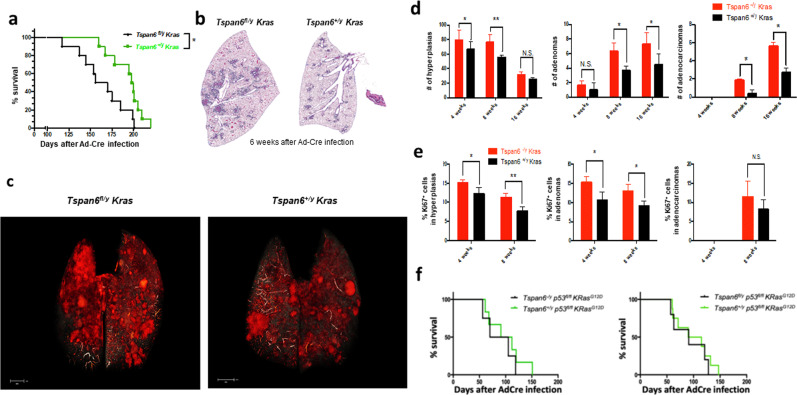
TSPAN6 has tumor cell-intrinsic functions. **a** Conditional *Tspan6* deletion significantly decreases survival (Kaplan Meier blot) in lung cancer-bearing *Tspan6*^*fl/y*^ *Kras*^*G12D*^ mice as compared to *Tspan6*^*+/y*^ *Kras*^*G12D*^ control littermates. *n* = 10 per group. *P < 0.05 (Log-rank test). **b** Representative H&E-stained histological sections of both cohorts 6 weeks after Ad-Cre inhalation. Magnifications ×5. **c** Representative microCT images to detect lung tumors in *Tspan6fl/y* *Kras*^*G12D*^ and *Tspan6+/y* *Kras*^*G12D*^ littermates 8 weeks after Ad-Cre inhalation. **d** Quantification of hyperplasic regions, adenomas and adenocarcinomas in lungs from *Tspan6*^*fl/y*^ *Kras*^*G12D*^ (*n* = 5) and control *Tspan6*^*+/y*^ *Kras*^*G12D*^ (*n* = 5) littermates at the indicated time points following Ad-Cre inhalation. Data are shown as means ± s.e.m. *P < 0.05; **P < 0.01; N.S. = not significant (Student’s *t* test). **e** Quantification of % Ki67^+^ cells in hyperplasic regions, adenomas, and adenocarcinomas 4 and 8 weeks post Ad-Cre infection. Data are shown as mean ± s.e.m. *P < 0.05; **P < 0.01; N.S. = not significant (Student’s *t* test). **f** *p53* ablation in *Kras*^*G12D*^ lung tumors abrogates the survival advantage conferred by *Tspan6* expression. Kaplan Meier survival plots are shown for whole-body *Tspan6*^*-/y*^ *p53*^*fl/fl*^ *Kras*^*G12D*^ mice (*n* = 4) and *Tspan6*^*+/y*^ *p53*^*fl/fl*^ *Kras*^*G12D*^ (*n* = 6) littermates, and conditional *Tspan6*^*fl/y*^ *p53*^*fl/fl*^ *Kras*^*G12D*^ mice (*n* = 5) and their respective *Tspan6*^*+/y*^ *p53*^*fl/fl*^ *Kras*^*G12D*^ (*n* = 8) littermates. A Log-rank test was used for statistical analysis.

Profiling *Tspan6* mRNA expression in the lung revealed transcripts in alveolar pneumocytes bronchial epithelium, endothelial cells, as well as resident macrophages (not shown). To therefore test whether the effects of Tspan6 on *Kras*^*G12D*^-induced lung cancer are cell autonomous to the transformed lung epithelium, we generated mice that carry a *Tspan6*^*floxed*^ allele. Cre-mediated recombination results in excision of Exons 2–6 and a shift in the reading frame (Supplementary Fig. 6a, b). Female *Tspan6*^*flox/flox*^ mice were then crossed to male *Lox-Stop-Lox-Kras*^*G12D*^ animals to generate male *Tspan6*^*fl/y*^ *Kras*^*G12D*^ and *Tspan6*^*+/y*^ *Kras*^*G12D*^ mice. In this model system, Ad-Cre inhalation results in concurrent deletion of *Tspan6* in the cells that express oncogenic *Kras*^*G12D*^ in the lung (Supplementary Fig. 6c). Selective loss of *Tspan6* in the *Kras*^*G12D*^-expressing lung epithelial cells again resulted in significantly reduced survival and increased tumor burden as compared to *Tspan6*-expressing *Kras*^*G12D*^ littermates (Fig. 6a–c). *Tspan6*^*fl/y*^ *Kras*^*G12D*^ mice also harbored significantly more hyperplastic lesions and increased numbers of adenomas and adenocarcinomas as compared to their *Tspan6*-expressing littermates (Fig. 6d). Western blotting of tumor tissue confirmed efficient loss of Tspan6 in *Tspan6*^*fl/y*^ *Kras*^*G12D*^ mice (Supplementary Fig. 5d). Similar to the whole-body mutant mice, cell proliferation was significantly enhanced in hyperplastic regions and adenomas from the conditional *Tspan6*^*fl/y*^ *Kras*^*G12D*^ mice (Fig. 6e). There were no significant differences in numbers of apoptotic cells (detected by cleaved caspase 3), intratumoral CD3^+^ T cells, intratumoral FoxP3^+^ regulatory T cells, and tumor angiogenesis as detected by CD31 immunostaining (Supplementary Fig. 7). In conclusion, our genetic data show that Tspan6 exerts a cell autonomous function in suppressing *KRas*^*G12D*^-driven initiation and progression of lung cancer.

Finally, we asked the question of whether the survival advantage conferred by *Tspan6* expression in *Kras*^*G12D*^ transformed cells is dependent on p53 using *p53*^*floxed*^ mice. Using either whole-body *Tspan6* knockouts or *Tspan6*^*floxed*^ alleles (Fig. 6f), tumor-specific deletion of *p53* abolished the protective effects of *Tspan6* expression on *Kras*^*G12D*^ transformed cells. In conclusion, our data indicate that Tspan6 exerts a cell autonomous function in suppressing *Kras*^*G12D*^-driven initiation and progression of lung cancer, and Tspan6’s tumor suppressor function is p53 dependent.

### *TSPAN6* expression stratifies survival in human lung and pancreatic cancer

**Fig. 7:**
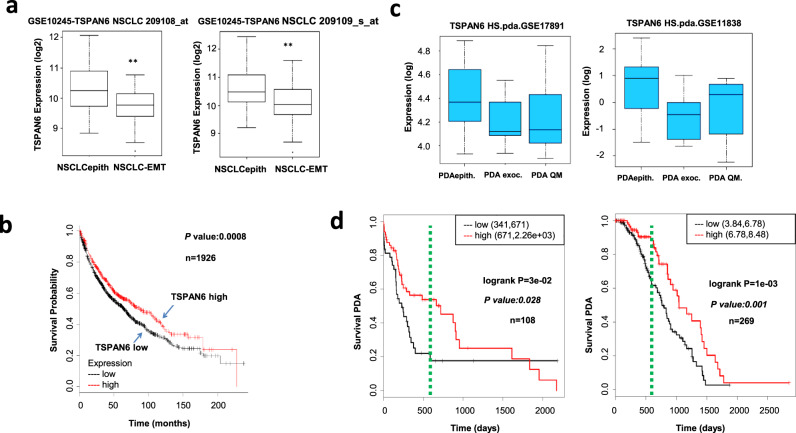
Low *TSPAN6* expression is correlated with poor survival in human lung and pancreatic cancer patient survival with mesenchymal signatures. **a** *TSPAN6* expression is significantly reduced in lung cancer (NSCLC) cancers from the GSE10245 cohort with an EMT signature ((NLCLC-EMT) versus an epithelial (NLCLCepith) signature (**p < 0.01)). The *TSPAN6* probes used are indicated. **b** Low *TSPAN6* expression levels (Affymetrix probes) predict poor overall survival of lung cancer patients from this cohort. Survival analysis was calculated using a log-rank test. Data presented were obtained using KM plotter. *P* values (log-rank test) and total numbers of patients with either low (black) or high (red) *TSPAN6* expression are indicated. **c** *TSPAN6* expression is significantly reduced in pancreatic adenocarcinoma (PDA) cancers with an EMT signature (PDA-exoc and PDA-QM) relative to those with an epithelial signature (PDA-epith) in the GSE17891 and GSE11838 cohorts. **d** Low *TSPAN6* expression is correlated with poor survival in pancreatic ductal carcinoma patients from these cohorts (data presented as in b). Green bar-*TSPAN6* low expression identifies PDA patients with advanced disease and early relapse with lethal outcome (18–24 months). NSCLCepith Non-Small Cell Lung Cancer epithelial subclass, NSCLCmes Non-Small Cell Lung Cancer mesenchymal subclass [36]; PDAepith Pancreatic Ductual Adenocarcinoma epithelial subclass, PDAexocrine Pancreatic Ductual Adenocarcinoma exocrine subclass, PDA-QM Pancreatic Ductual Adenocarcinoma quasi-mesenchymal subclass [37].

Subgroups of Non-Small Cell Lung Cancer (NSCLC) were identified based on EMT and epithelial-expression signatures with differing therapy responses [35, 36]. As our results showed that depletion of TSPAN6 in epithelial cells leads to an EMT and tumor progression, we asked whether *TSPAN6* expression could be used to differentiate between EMT and epithelial subgroups of NSCLC tumors. *TSPAN6* expression indeed differentiated between the EMT and epithelial NSCLC tumor subgroups, with high *TSPAN6* expression being associated with an epithelial signature (Fig. 7a), which correlates with better therapy response and overall patient survival [36]. We then asked whether *TSPAN6* expression in NSCLC tumors predicts patient survival. Survival analysis conducted for 1926 NSCLC patients revealed a significant correlation (*p* = 0.0008) between low *TSPAN6* tumor expression and reduced patient survival (Fig. 7b).

Similarly, pancreatic adenocarcinomas have been classified based on their EMT and epithelial-expression signatures [37]. We found that low expression of *TSPAN6* correlated with pancreatic adenocarcinomas with an EMT signature (Panc-exoc and Panc-Qm) relative to those with an epithelial signature (Panc-epith) in two pancreatic cancer cohorts [37, 38] (Fig. 7c). Importantly, low *TSPAN6* expression also significantly correlated with poor survival in these pancreatic adenocarcinoma cohorts (*p* = 0.028 for GSE17891 and *p* = 0.001 for GSE11838) (Fig. 7d). Together these data indicate that low *TSPAN6* expression levels correlate with lung and pancreatic cancer EMT signatures and also with poor survival in these cancer patients.

## Discussion

**Fig. 8:**
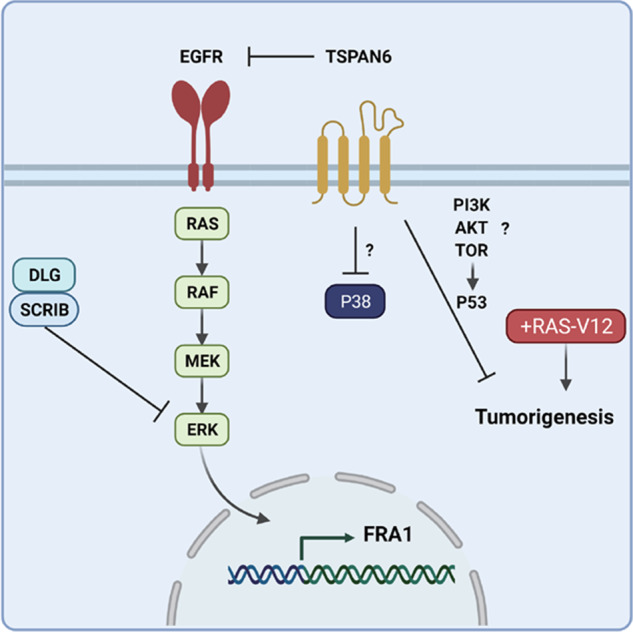
Model for the role of TSPAN6 in the regulation of signaling pathways in tumorigenesis. We show in this study that depletion of TSPAN6 cooperates with oncogenic RAS in tumorigenesis and that the tumor suppressor role of TSPAN6 requires P53. We show that TSPAN6 binds to and inhibits EGFR activation and thereby inhibits RAS activation. TSPAN6 does not bind to SCRIB or DLG1, suggesting that they act in parallel to each other in the regulation of the EGFR-RAS-ERK pathway, with TSPAN6 regulating EGFR and SCRIB/DLG1 regulating ERK. TSPAN6 also inhibits P38 activation by an unknown mechanism. TSPAN6 may be required for the activation of the tumor suppressor P53 by a pathway involving the activation of PI3K-AKT-TOR. Additionally, TSPAN6 alters epithelial cell morphology with its depletion leading to an EMT, which may further activate RAS-ERK signaling through SCRIB/DLG1 impairment. “?”, indicate that the potential involvement of TSPAN6 in these pathways needs to be confirmed.

Our study has revealed a novel role for the Tetraspanin TSPAN6 as a tumor suppressor in Ras-driven cancer (Fig. 8). We show that in normal human mammary epithelial cells, knockdown of *TSPAN6* cooperates with *H-RASV12* to drive invasive properties, similarly to knockdown of the cell polarity regulators SCRIB and DLG1. In mouse orthotopic transplantation experiments, *Tspan6* knockdown promotes tumorigenesis of *H-RasV12*-transformed mouse mammary epithelial (EpRas) cells, and overexpression of *TSPAN6* abrogates the tumorigenic potential of *K-Ras* activating mutant human pancreatic cancer cells. Moreover, whole-body and lung-specific knockout of *Tspan6* cooperates with *Kras*^*G12D*^ to promote lung cell tumors, by promoting cell proliferation, an EMT and invasive/metastatic properties. Moreover, Tspan6’s tumor suppressor function in *Kras*^*G12D*^-driven lung cancer in the mouse model is p53 dependent. Importantly, low *TSPAN6* expression was correlated with an EMT signature and poor survival of human patients with non-small cell lung and pancreatic cancers, indicating that TSPAN6 functions as a tumor suppressor in human epithelial cancers. Mechanistically, TSPAN6 binds to the EGFR to inhibit EGFR-RAS-ERK signaling.

Our data showing knockdown of *TSPAN6* phenocopies the knockdown of *SCRIB* and *DLG1* in cooperation with *H-RasV12* is consistent with our observations that *TSPAN6* knockdown induces cell morphology changes leading to an EMT in cooperation with oncogenic RAS. Our previous studies in *Drosophila* have revealed that the TSPAN6 ortholog, Tsp29Fb, regulates Dlg1 protein localization and genetically interacts with *scrib* in epithelial development [14], and whether TSPAN6 also affects DLG1/SCRIB localization/function in mammalian cells is of interest to determine. In *Drosophila*, various Tetraspanins have been linked to the assembly or function of the Septate Junction [39, 40], which functions similarly to mammalian Tight Junctions in epithelial barrier formation [41]. Interestingly, the *Drosophila* Tetraspanin protein, Tsp2A, regulates the stability of the cell polarity protein, atypical protein kinase C (aPKC), which is a regulator of Hippo-Yki and JAK-STAT signaling in intestinal epithelial cells, thereby limiting cell proliferation [40]. Whether Tsp29Fb/TSPAN6 also regulates aPKC to mediate its tumor suppressor effects remains to be determined.

Mechanistically, our genetic and biochemical experiments show that TSPAN6 modulates EGFR-RAS-ERK signaling (Fig. 8). Moreover, TSPAN6 can bind to the EGFR suggesting that it directly negatively regulates EGFR phosphorylation and signaling. Indeed a recent study has shown that TSPAN6 controls the production of the extracellular vesicles containing the transmembrane form of TGF-alpha, a EGFR ligand, and thereby downregulates EGFR signaling [42]. In this newly described mechanism, TSPAN6 is physically linked to TNF-alpha via the adapter protein syntenin-1, and upon TSPAN6 downregulation increased production of extracellular tm-TGF-alpha occurs leading to increased EGFR signaling [42]. Although our findings show that *TSPAN6* knockdown phenocopies *DLG1* and *SCRIB* knockdown, and our analysis in *Drosophila* has linked Tsp29Fb to Scrib/Dlg1 regulation/function [14], and that Scrib/Dlg1 are known to regulate Ras signaling in *Drosophila* and mammalian cells [11, 30, 43, 44], since TSPAN6 does not bind to DLG1 or SCRIB, it is likely that these proteins regulate EGFR-RAS signaling by parallel pathways, with TSPAN6 functioning at the level of the EGFR by binding to it and inhibiting its activation, and SCRIB/DLG1 directly binding to and negatively regulating ERK activation [43]. However, it is possible that knockdown of Tsp29Fb/TSPAN6 may also deregulate RAS-ERK signaling indirectly through inducing an EMT and impairing SRIB/DLG1 function.

Whether Tsp29Fb/TSPAN6 regulates other signaling pathways needs to be further explored; however the decrease in phospho-P38 observed upon *TSPAN6* overexpression, suggests that it also negatively regulates the P38-stress response pathway (Fig. 8), a JUN-kinase-related pathway that cooperates with oncogenic Ras in tumorigenesis in *Drosophila* and mammalian systems [45, 46]. Furthermore, how the tumor suppressor role of TSPAN6 in RAS-driven tumorigenesis is linked to P53 function requires further analysis. P53 is a key tumor suppressor that is mutated in ~50% of human cancer, which functions by inhibiting cell cycle progression, promoting senescence and inducing cell death [47–49]. Since high levels of RAS signaling can also induce cell cycle arrest and senescence [7, 8], it is possible that P53 contributes to these tumor-suppression mechanisms and TSPAN6 might be involved in P53 activation. Interestingly, another tetraspanin, CD9, induces senescence by inducing P53 via the PI3K-AKT-TOR pathway [50]. Intriguingly, TSPAN6 overexpression led to an increase in phospho-AKT and phospho-TOR suggesting that it may also function similarly to CD9 in inducing P53 activity through the PI3K-AKT-TOR pathway (Fig. 8). Further analysis is required to confirm and further elucidate the potential regulation of P38 and PI3K-AKT-TOR signaling and P53 activity by TSPAN6.

In *Drosophila*, apical-basal cell polarity mutants or deregulation of cytoskeletal proteins (such as Src, Rac1, RhoGEF2), result in epithelial cells undergoing cell morphology changes and becoming migratory, resembling an EMT in mammalian cells [25], and knockdown of the *TSPAN6* ortholog, *Tsp29Fb*, in *Drosophila* affects Dlg1 localization, enhances *scrib* mutant cell polarity-impaired phenotypes and promotes invasive overgrowth together with oncogenic *Ras* [14]. Importantly, knockdown of *Tspan6* was sufficient to trigger an EMT in Ras-transformed mouse mammary epithelial cells and in in vivo tumor experiments we also observed an EMT, as defined by altered Vimentin and E-Cadherin expression, in *Tspan6* knockdown cells. Moreover, induction of *TSPAN6* in human pancreatic cancer cells inhibits EGFR-induced expression of the EMT markers Vimentin and N-Cadherin, as well as the critical EMT transcription factor Slug, thereby linking TSPAN6 to the inhibition of EGFR-mediated EMT in human tumor cells. Together, these observations in *Drosophila* [14] and mammalian cells (this study) suggest that TSPAN6 plays a role in regulating apico-basal cell polarity and controlling cellular architecture. Whether these effects on epithelial architecture modulate EGFR-RAS-ERK activation (perhaps through altering SCRIB/DLG1 function) or affect other signaling pathways to contribute to the tumor suppressor function of Tsp29Fb/TSPAN6 needs to be examined. Considering our results that TSPAN6 had no apparent effect on EGFR-induced proliferation but markedly altered invasive behavior in response to EGF, TSPAN6-regulated cell polarity and an EMT could conceivably explain why the modulation of TSPAN6 expression affects metastatic tumor spread.

In summary, we have shown that TSPAN6 binds to the EGFR and inhibits EGFR-RAS-ERK signaling. Furthermore, our results show that overexpression of *TSPAN6* impairs cell proliferation and invasion of pancreatic tumor cells in vitro and abrogates tumor growth and metastatic spread in orthotopic tumor implants in vivo. Conversely, knockdown of human *TSPAN6* in human mammary epithelial cells enhances the oncogenic *RAS*-driven invasive phenotype, and knockdown of mouse *Tspan6* enhances cell proliferation and in vivo tumor growth and metastases of oncogenic *Ras*-transformed mouse epithelial cells. Importantly, by generating *Tspan6* whole-body mutant mice and a conditional *Tspan6*^*floxed*^ allele, we provide definitive evidence that loss of *Tspan6* markedly enhances the initiation as well as malignant progression of oncogenic *Kras*-driven lung cancer in a tumor cell autonomous manner. Additionally, our results indicate that loss of *Tspan6* results in enhanced epithelial proliferation and altered epithelial architecture implicated in cell morphology, migration and tumor invasion/metastasis. Thus, TSPAN6 constitutes an evolutionary conserved bona fide suppressor of oncogenic RAS-driven tumor growth and invasion/metastasis in human pancreatic cancer cells, human and mouse mammary epithelial cells and mouse lung tumors. Moreover, our analysis of human lung and pancreatic cancer cohorts shows a correlation of low *TSPAN6* expression with cancers with an EMT signature and with poor patient survival. In addition to lung and pancreatic cancer, mining of various databases, including TCGA, revealed mutations, deletions, and copy number variations of *TSPAN6* in multiple human cancers (not shown). A recent study has revealed that TSPAN6 also plays a tumor suppressor role in colorectal cancer [42], but whether TSPAN6 has a tumor suppressor role in additional cancer types, apart from pancreatic, lung and colorectal, needs to be explored in future experiments.

## Materials and methods

### Mammary and pancreatic cell line analysis

See Supplementary information.

### Generation of *Tspan6* mutant mice

Whole-body *Tspan6* mutant mice were generated by DeltaGene. Briefly, a targeting vector was inserted into exon 2 of the murine *Tspan6* locus. The linearized construct was electroporated into embryonic stem (ES) cells derived from the 129/OlaHsd mouse sub-strain. Correctly targeted ES cell clones were confirmed by Southern blotting and used to generate chimeric mice. Germline transmitted F1 mice were backcrossed to C57BL/6 females. The following primers were used for genotyping giving a mutant band of 405 bp and a 260 bp wild type band: endogenous forward primer TGTGATCAAGGACTCAAGCTTGTAC; the Neo primer GGGTGGGATTAGATAAATGCCTGCTCT; and the endogenous reverse primer CTTACTCACCAGTTTCAGCATCCAG.

For the generation of conditional *Tspan6*^*floxed*^ mice we performed recombineering to introduce *loxP* sites, and positive and negative selection markers, into BAC DNA by homologous recombination. BAC clones were ordered from bacpac.chori.org/vectorsdet.htm containing the region of interest of the *Tspan6* gene. The final targeting construct was electroporated into A9 ES cells which were screened for correct recombination events via PCR. For PCR screening the following primers were used:

Neo/Short arm primers—product size 998:

1. Forward 5′ TGGACGTAAACTCCTCTTCAGACCT 3′ (*T*_m_ = 61C)
2. Reverse 5′ CCCTGTCTTAGTTGGCCCTAACAGTA 3′ (*T*_m_ = 61C)
  Short arm/genomic region primers - product size 1052
3. Forward 5′ TCCTTATCGCCTCCAAGGAAAGGAT 3′ (*T*_m_ = 61C)
4. Reverse 5′CAGAGGCAGGTAGATCTTCAGATCTTC 3′ (*T*_m_ = 60C)

Conditional *Tspan6*^*fl/fl*^ and whole-body *Tspan6* mutant mice were backcrossed for at least ten generations onto a C57BL/6J background and then crossed to *LSL-K-Ras*^*G12D*^ mice to generate *Tspan6*^*fl/y*^ *LSL-K-Ras*^*G12D*^ *and* *Tspan6*^*+/y*^ *LSL-K-Ras*^*G12D*^ mice and their respective Tspan6 expressing littermate control cohorts. Mouse genotypes were determined by PCR and, if required for confirmation, by DNA blot analysis. In all experiments, only age and sex-matched littermate mice were used. All mice were maintained according to the ethical animal license protocol complying with the Austrian and European legislation.

### Induction of lung cancer

Inhalation of mice with *Ad-Cre* viruses was performed as previously reported [51]. In brief, experimental animals were anesthetized with 10% Ketasol/Xylasol and placed on a heated pad. An Ad-Cre-CaCl2 precipitate was produced by mixing 60 μl MEM, 2.5 μl Ad-Cre (1010 pfu/ml; University of Iowa, Gene Transfer Vector Core Iowa, USA) and 0.6 μl CaCl2 (1 M) for each mouse and incubated for 20 min at room temperature (21–22 °C).

### Histology, immunohistochemistry, and lung/tumor ratio quantification

Histological and immunohistochemical analysis of lung tumors was performed as previously described [51]. Briefly, 2 µm sections from at least 3 different planes of the lung were cut and stained with H&E. Sections were scanned using a Mirax slide scanner and lung/tumor areas automatically scored by an algorithm programmed and executed using the Definiens software suite and visually controlled in a blinded way. Immunohistochemistry staining was done using an automatic staining machine (Leica Bond3) or manually processed. Sections were dehydrated and antigenic epitopes retrieved using a 10-mM citrate buffer and microwaving for 10 min Specimens were then incubated with anti-Foxp3 (eBioscience, 13-5773), anti-CD3ε (Santa Cruz, 101442), anti-Ki67 (Novocastra), anti-CD31 (Abcam, ab28364), and anti-cleaved Caspase 3 (Cell signaling, 9661). Primary antibody staining was detected by peroxidase-conjugated anti-rabbit IgG. Positive cells were counted on 20 randomly chosen tumor areas at ×400 magnifications in a double-blinded fashion. Images were captured with a Zeiss AxioImager Z1. Quantitative analysis was performed using HistoQuestTm software (TissueGnostics GmbH, Vienna, Austria, www.tissuegnostics.com). These experiments were independently replicated, with biological and technical replicates as detailed in the legend of the corresponding figure, with similar results.

### MicroCT scanning

Formaldehyde perfused and fixed lungs were stained in a solution of 1% elemental iodine and 2% potassium iodide in distilled water for three days. After staining, specimens were rinsed mounted in plastic tubes for microCT scanning. Lungs were scanned using a SCANCO µCT 35 (SCANCO Medical AG, Brüttisellen, Switzerland) with a source energy of 70keV and an intensity of 114 µA using a 0.2 mm copper filter. Projection images were recorded with an angular increment of 0.36°. Reconstructed microCT slices measured 1024 × 1024 pixels (voxel size = 20 µm). Image stacks were imported into Amira^®^ 5.3 (Visualization Sciences Group, Mérignac Cedex, France) and filtered with a 3D median filter (3 × 3 × 3 kernel). For segmentation of lung and tumor tissue, specific attenuation thresholds were used. For discriminating background from lung tissue an X-ray attenuation value of *µ* = 0.3987 and for discriminating lung tissue from tumor tissue an attenuation value of *μ* = 1.2776 was used, respectively. Based on segmentation, lung tissue and tumor volumes were calculated. These experiments were independently replicated, with biological and technical replicates as detailed in the figure legend, with similar results.

### Human cancer data and statistical analysis

See Supplementary information.

## Supplementary information

Supplementary Information and Figures
