# Correction: TSPAN6 is a suppressor of Ras-driven cancer

**DOI:** 10.1038/s41388-022-02265-2

**Published:** 2022-03-15

**Authors:** Patrick O. Humbert, Tamara Zoranovic Pryjda, Blanka Pranjic, Andrew Farrell, Kohei Fujikura, Ricardo de Matos Simoes, Rezaul Karim, Ivona Kozieradzki, Shane J. F. Cronin, G. Gregory Neely, Thomas F. Meyer, Astrid Hagelkruys, Helena E. Richardson, Josef M. Penninger

**Affiliations:** 1 Department of Biochemistry & Genetics, School of Molecular Sciences, La Trobe University, Bundoora, VIC 3086 Australia; 2 Institute of Molecular Biotechnology of the Austrian Academy of Science, Dr. Bohrgasse 3, 1030 Vienna, Austria; 3 Max Planck Institute for Infection Biology, Charite Platz 1, 10117 Berlin, Germany; 4 Department of Medical Genetics, Life Sciences Institute, University of British Columbia, Vancouver, BC Canada; 5 Center for Integrative Bioinformatics Vienna, Max F. Perusetz Laboratories, Dr.-Bohr-Gasse 9/6, A-1030 Vienna, Austria; 6 Dr. John and Anne Chong Lab for Functional Genomics, Charles Perkins Centre, School of Life and Environmental Sciences, The University of Sydney, Sydney, NSW 2006 Australia; 7 Laboratory of Infection Oncology, Institute of Clinical Molecular Biology, Christian Albrecht’s University of Kiel and University Hospital Schleswig Holstein—Campus Kiel, Kiel, Germany

**Keywords:** Cell polarity, Growth factor signalling, Cancer genetics

Correction to: *Oncogene* 10.1038/s41388-022-02223-y, published online 19 February 2022

In this article, the sentence “In this newly described mechanism, TSPAN6 is physically linked to TNF-alpha via the adapter protein syntenin-1, and upon TSPAN6 downregulation increased production of extracellular tm-TNF-alpha occurs leading to increased EGFR signaling [42]”. Should have read “In this newly described mechanism, TSPAN6 is physically linked to TGF-alpha via the adapter protein syntenin-1, and upon TSPAN6 downregulation, increased production of extracellular tm-TGF-alpha occurs leading to increased EGFR signaling [42]”.

The original article has been corrected.

