# Supplementary material for: TSPAN6 is a suppressor of Ras-driven cancer
Source: Oncogene. 2022 Feb 19;41(14):2095–105. doi: 10.1038/s41388-022-02223-y (PMC8975741; doi:10.1038/s41388-022-02223-y)
Supplement: Supplementary file 1 — Supplementary Information and Figures [file 41388_2022_2223_MOESM1_ESM.pdf]

# Supplementary online data

Supplementary Materials and Methods

Supplementary Figure legends

Supplementary References

Supplementary Figures

# Supplementary Materials and Methods

**Generation of *TSPAN6, SCRIB* and *DLG1* knockdown, and *H-RASV12* MCF10A cell lines and culturing conditions.**

*TSPAN6* and *SCRIB* shRNA gene-set constructs were in the shERWOOD UltramiR shRNA lentiviral vector (puro, GFP), obtained from Transomic (Huntsville, AL, USA).

*DLG1* constructs were generated using the primers below in the pSuper-retro vector (puro). sh-RNA pSuper-retro *shDLG1* 5B was constructed using primers a5 and b5 and pSuper-retro *shDLG1* 6E was constructed using primers a6 and b6 (Galea et al unpublished).

*shDLG1*a5

GATCCCCGTATCAGGATGAAGATACATTCAAGAGATGTATCTTCATCCTGATACTTTTTG
GAAA

*shDLG1*b5

AGCTTTTCCAAAAAGTATCAGGATGAAGATACATCTCTTGAATGTATCTTCATCCTGATA
CGGG

*shDLG1*a6

GATCCCCGTCAGGGACTGAACTTCAATTCAAGAGATTGAAGTTCAGTCCCTGACTTTTT
GGAAA

shDLG1b6

AGCTTTTCCAAAAAGTCAGGGACTGAACTTCAATCTCTTGAATTGAAGTTCAGTCCCTGA
CGGG

The *H-RASV12* vector used was *MSCV-H-RasV12-IRES-Cherry* [4]. High-Ras expressing cells were selected by FACS sorting of high mCherry expression and the low-Ras expressing cells were selected on the basis of low-mCherry expression.

MCF10A cells were cultured and transduced as previously described [3, 4]. Briefly, HEK293T cells, cultured in DMEM (Invitrogen) and 10% FCS, were transfected with the shRNA and/or *H-RASV12* vectors using the manufacturer's instructions. Following transfection, the media was replaced with complete MCF10A media and the following day the media containing the lentivirus was used to transduce MCF10A cells. Approximately 2 days following transfection, the cells were selected in 2μg/ml puromycin for one week and then sorted on a FACStar flow cytometer for either GFP or mCherry (Becton Dickinson, Franklin Lakes, NJ).

**MCF10A Scratch wound healing assay.**

MCF10A cells containing stable knockdown of *SCRIB, DLG1* or *TSPAN6* +/- *H-RAS*$^{V12}$ were generated as previously described [4]. 4 x $10^4$ MCF10A cells were plated per well in a 24 well plate and the cells were allowed to reach confluency over 48 hours. On Day 3, the cells were washed with DPBS (Invitrogen) and then placed in starvation media (media minus epidermal growth factor). The following day, the starvation media was removed and DPBS was added to each well. A "wound" was then created using a sterile P200 pipette tip, and 2 more washes with DPBS. Regular MCF10A media was then used, supplemented with 1 μg/ml mitomycin C (Sigma). The wound area was then imaged over a 24-hour time period using the Lionheart FX Imager (Biotek). The wound area was calculated using ImageJ software and the MRI Wound Healing Tool (http://dev.mri.cnrs.fr/projects/imagej-macros/wiki/Wound_Healing_Tool). These experiments were independently replicated, with biological and technical replicates, as detailed in the figure legend, with similar results.

**3D invasion assay.**

Three-dimensional MCF10A organotypic cultures were performed using the 'overlay method' as previously described [3]. Following 7 days of growth, each well was imaged multiple times using the Leica DMi1 microscope (Leica). The number of invasive acini was then counted after imaging via manual analysis in a blinded fashion. These experiments were independently

replicated, with biological and technical replicates, as detailed in the figure legend, with similar results.

**H-Ras transformed mouse epithelial cells.**

EpRas cells were derived from the mouse mammary EpH4 epithelial cell line expressing the viral *H-Ras*$^{V12}$ oncogene as previously described [6, 7] and cultured at 37°C, 5% $CO_2$ and 95% humidity in DMEM (Dulbecco´s modified ALPHA MEM-medium; Invitrogen) containing 10% FCS (Fetal Calf Serum; Invitrogen) and penicillin/streptomycin (Invitrogen, 100 U/ml penicillin and 100 µg/ml streptomycin). Stable knockdown of TSPAN6 in mouse mammary cell lines was obtained using SIGMA transduction particles (Mission, SHCLNV-NM_019656). Two different hairpins targeting TSPAN6 were selected containing the following sequences:

*Tspan6 shRNAm1*:

CCGGCCGGTCATTACTTGTTTGAAACTCGAGTTTCAAACAAGTAATGACCGGTTTTTG

*Tspan6 shRNAm2*:

CCGGGCGATGTTTCTGACACTCATTCTCGAGAATGAGTGTCAGAAACATCGCTTTTTG

Serum-free three-dimensional cultures of EpH4 cells, EpRas cells, and their derivatives were performed as described [6] with modifications. Briefly, cells were mixed with collagen (3–4 mg/ml; #40236; Becton Dickinson) at 0°C (final collagen concentration 1.5 mg/ml) and 100-µl droplets containing between 2,000 and 3,000 cells were dispensed into 17-mm wells. After 45 min of polymerization, mammary epithelial cell media (PromoCell) supplemented with TGFβ, insulin, dexamethasone, isoproterenol and bovine pituitary extract (PromoCell) was added. Collagen gels were fixed in 4% PFA/PBS after 7 days, washed 3 × with PBS and stained for ZO-1 and vimentin expression (rabbit anti–ZO-1 (61–7300; Zymed Laboratories), monoclonal anti–mouse vimentin, Vim-13.4 (V-2258; Sigma-Aldrich). After staining, gels were washed with PBS, fixed with 4% PFA/250 mM HEPES, and counterstained with DAPI. Images were taken using a Carl Zeiss LSM 410 UV confocal laser scanning microscope. For uptake of $^3$H-thymidine EpRas cells were cultured to 80% confluency and serum starved over-night. After splitting, cells were counted and 50,000 cells/ well were transferred into a 96-well plate. After adhering, 0.1ml of 0.1 mCi/mL $^3$H-thymidine was added into each well. Cells were incubated with $^3$H-thymidine for 8 hours and uptake determined using a scintillation counter.

For Boyden chamber migration assays, cells were prepared as above. Invasion was assayed after 24 hours. All experiments were repeated at least 3 times. For induction of tumors and metastases in mice, EpRas cells transfected with scrambled shRNA or two different shRNAs to knockdown *Tspan6* (*Tspan6 shRNAm1* and *Tspan6 shRNAm2*) were orthotopically injected into the 3rd and 4th mammary gland fat pad (200,000 cells per site in 50µl PBS) of Ketamine anaesthetized nu/nu females (Charles River Wiga, GmbH). Primary tumor growth was weekly measured with a caliper in mm. Mean tumor volume was calculated using two measured diameters according to the formula: $r1^2 \times \frac{r2}{2}$ . For the experimental metastasis assays, EpRas cells ($5 \times 10^5$ cells re-suspended in 100 µl PBS) were injected into the tail vein of *nu/nu* mice. Morbidity was assessed daily and upon detection of first moribund mice the entire cohort was sacrificed. Multiple tissues (with the exception of brain and bone) were isolated, sectioned, and processed for histopathological detection of metastases. For *in situ* immunohistochemical detection of E-cadherin, tumors were harvested and embedded in paraffin. Following de-paraffination and antigen retrieval using microwaving in 0.01M citric buffer (pH6.0) for 15 min, endogenous peroxidase was blocked by a solution containing 23.5 mL methanol, 1.5 mL $H_2O_2$ and 25 mL $H_2O$. Slides were then washed, blocked in 10% goat serum/PBS for 1 hour at RT, and specimen incubated with antibodies against E-cadherin (BD Transduction Labs #610404, 1:1000) in blocking solution O/N. Sections were then incubated with a secondary biotinylated anti-rabbit antibody (DAKO EnVision) for 30 min, treated with DAB reagent to visualize Ab binding and counter-stain with hematoxylin (1:3 in $H_2O$ distilled).

For EMT marker mRNA expression analysis, stable Tspan6-mCherry expressing MIA-PACA-2 or PANC1 clones were either left un-induced or induced with doxycycline for 48 hours. The cells were then serum starved for additional 24 hours in presence or absence of doxycycline and/or erlotinib (2µM). Cells were then either left unstimulated or stimulated with recombinant human EGFα (1 ng/ml) for 8 hours at 37°C. Total RNA was isolated using TRIzol (Invitrogen) followed by cleanup using the RNeasy Mini Protocol (Qiagen). Total RNA (0.2 µg) was reverse transcribed using SuperScript III (Invitrogen) and oligo dT primers (Promega). Triplicate PCR reactions were performed with 20 pmol of gene-specific primers and iQ SYBR Green Supermix (Bio-Rad) using PCR conditions and primers as described previously [9]. Threshold cycle

numbers (Ct) were determined with C1000 Thermal Cycler (Bio-Rad) and the relative quantities of mRNA per sample were calculated using the ΔΔCt method with *GAPDH* as the calibrator gene. The relative levels of mRNA were determined by setting the mRNA expression level of the first sample as previously described [9]. These experiments were independently replicated, with biological and technical replicates, as detailed in the figure legend, with similar results.

**Generation of TSPAN6 over-expressing human pancreatic cancer cells.**

*K-Ras* mutant human pancreatic cancer cell lines were chosen based on low *TSPAN6* mRNA expression. The cells were cultured in DMEM supplemented with 10% FBS, 2mM L-glutamine, and 100 U/ml penicillin and 100 µg/ml streptomycin at 37°C with 5% $CO_2$. A human *TSPAN6* ORF cDNA clone (NM_003270.2) was obtained from GeneCopoeia and sequence verified at all cloning steps. For inducible expression of *TSPAN6*, the *Tet-ON 3G* inducible expression system with *mCherry* (Clontech) was used according to manufacturer's instructions. In short, the cells were transfected with the regulator vector *pCMV-Tet3G* and selected with G418 (Sigma-Aldrich). The cells were then transfected with human *TSPAN6*-cloned response vector *pTRE3G-mCherry* and selected with puromycin (InvivoGen). Finally, cells were treated with doxycycline (10 ng/ml, Sigma-Aldrich) for 48-72 hours and high mCherry expressing cells were FACS sorted. Stable cell lines were maintained in the above mentioned medium supplemented with one-third of the concentrations of G418 and puromycin used initially for selection to maintain selection pressure. Cultured cells were then used for *in vitro* assays or orthotopically injected into the pancreas of *nu/nu* mice.

**Western blot analysis and antibody array**.

For Western blotting, polypeptides were resolved by SDS–polyacrylamide gel electrophoresis (SDS–PAGE) and transferred to a PVDF membrane (Bio-Rad). Immunodetection was achieved with anti-TSPAN6 (Sigma-Aldrich), anti-phospho EGFR Tyr1173 (Millipore), anti-total EGFR (Thermo Scientific), and anti-β-Tubulin (Sigma-Aldrich) antibodies. The proteins were visualized by a chemoluminescence reagent (Thermo Scientific). For the human phospho-kinase antibody array (R&D Systems), *TSPAN6*-expressing MIA-PACA-2 stable cells were either left untreated or induced with doxycycline (10 ng/ml) for an initial 48 hours. The cells

were then serum starved for an additional 24 hours in the presence or absence of doxycycline. The cells were left untreated or stimulated with recombinant human EGFα (20 ng/ml, Peprotech) for 5 minutes. 100 μg of cell lysate were added to the membrane and incubated overnight at 4°C with gentle agitation. Films were scanned and the intensity of the bands quantified using Image J. The relative amount was determined by calculating the ratio of each protein over that of the density measured for the housekeeping protein. In the case of the antibody array, samples without doxycycline were used as reference for each antibody. Ras activation was monitored in the same experimental set-up and EGFα stimulation conditions as above using an ELISA; 30 μg of cell lysate were added into each well and analyzed according to the manufacturer's instructions (Millipore). For each experimental condition, 6 wells were used. These experiments were independently replicated, with biological and technical replicates, as detailed in the figure legend, with similar results.

**Cell proliferation and migration assays.**

For proliferation, stable *TSPAN6-mCherry* expressing MIA-PACA-2 or PANC1 clones were either left untreated or induced with doxycycline for 48 hours. The cells were then serum starved for an additional 24 hours in the presence or absence of doxycycline. Cells were then either left unstimulated or stimulated with recombinant human EGFα (1 ng/ml) for 4 hours at 37°C. Proliferation was determined by BrdU labeling and analyzed via chemiluminescence according to the manufacturer's instructions (Roche). Cell migration assays were performed using a scratch assay or transwell invasion assay. Briefly, *TSPAN6*-expressing PANC1 stable cells were either untreated or induced with doxycycline for an initial 48 hours followed by serum starvation for an additional 24 hours in the presence or absence of doxycycline. Cells at 80-90% confluency were treated with mitomycin C (10 μg/ml, Sigma) for 2 hours in order to inhibit and exclude effects of cell proliferation. Confluent monolayers were then scratched and cell migration was monitored by microscopy 24 hours after the scratch. Cell invasion assays were carried out using 8μm transwell Boyden chambers (Corning). Collagen I (Thermo Scientific) was used as an extracellular matrix. Untreated or EGFα treated (0 ng/ml to 200 ng/ml) cells were placed into the top chamber. Fetal bovine serum (FBS, 0% or 10%) was placed into the bottom chamber as a chemoattractant. After overnight incubation at 37°C, the numbers of cells

that had migrated to the bottom of the chamber were counted using a hemocytometer. Experiments were carried out in triplicate. These experiments were independently replicated, with biological and technical replicates, as detailed in the figure legend, with similar results.

**Pancreatic tumor xenograft experiments.**

*TSPAN6*-mCherry expressing MIA-PACA-2 or PANC1 cells (5x$10^5$) were orthotopically injected into the pancreas of MF1-*Foxn1*$^{nu}$ nude mice (Harlan) as previously established (Kim et al. 2009). Whole-body bioluminescent imaging was performed using an IVIS100 system (Caliper LifeSciences). For TSPAN6 induction, mice were treated with doxycycline in both drinking water (2 mg/ml with 2% sucrose; Sigma-Aldrich) and food (625 mg/kg, Harlan Laboratories). Imaging was done at multiple time points after orthotopic injection. For histological analyses, paraffin-embedded tumor and normal tissue samples were prepared. Serial breast tumor, lung and liver sections were cut (5µm thickness), transferred to glass slides and left 1h at room temperature (RT) to dry. Normal skin, parathyroid and breast tissue samples were cut (6µm thick sections) and processed for hematoxylin & eosin (H&E) staining according to standard protocols. These experiments were independently replicated, with biological and technical replicates, as detailed in the figure legend, with similar results.

**Co-immunoprecipitation Analysis**

For co-immunoprecipitation with the anti-EGFR antibody, PANC1 and MIA-PACA2 cell lines were cultured in tissue culture flasks until they reach 80% confluency. Subsequently, each 1 x $10^8$ cells were washed with sterile PBS (pH 7.4). Co-immunoprecipitation was performed according to the Dynabeads Co-Immunoprecipitation Kit protocol (ThermoFisher) with minor modifications as described below. Briefly, EGFR antibody or Rabbit IgG antibody was coupled to M-270 Epoxy beads. Proteins were solubilized in protein extraction solution (1% Brij 98) and then supernatants were incubated with antibody-beads complex. Then, beads were washed with lysis buffer, and the eluted proteins were fractionated by SDS-PAGE and subjected to immunoblot analysis with an anti-TSPAN6 antibody (Abcam ab236883).

For co-immunoprecipitation studies with the FLAG-tagged TSPAN6 construct (pCMV6-TSPAN6), PANC1 and MIA-PACA2 cell lines were cultured in tissue culture flasks until they reach 70% confluency. Subsequently, these cells were transfected with pCMV6-FLAG-TSPAN6 constructs, using Effectene (Qiagen, Chatsworth, CA) according to the manufacturer's instructions. Each 5 x $10^7$ cells were washed with sterile PBS (pH 7.4). Co-immunoprecipitation was performed according to the Dynabeads Co-Immunoprecipitation Kit protocol (ThermoFisher) with minor modifications as described below. Briefly, the anti-FLAG antibody or the control IgG antibody were coupled to M-270 Epoxy beads. Proteins were solubilized in protein extraction solution (1% Brij 98) and then supernatants were incubated with antibody-beads complex. Then, beads were washed with lysis buffer, and the proteins binding to the M-270 Epoxy beads were collected using elution buffer. The immunoprecipitated proteins were fractionated by SDS-PAGE and subjected to immunoblot analysis with anti-EGFR (Abcam ab52894), anti-DLG1 (Anti-SAP97 Thermo Scientific PA1-741), anti-SCRIB (Cell Signaling Technology 4475S), or anti-FLAG (Thermo Scientific MA1-91878) antibodies. These experiments were conducted once.

**TSPAN6 correlation to EMT signature in NSCLC**.

Scoring of patient tumor samples based on an EMT and epithelial signature was performed based on NSCLC patient cohort analysis performed by Tan and colleagues [10]. *TSPAN6* expression between two patient groups of mesenchymal-like and epithelial-like samples was analysed. EMT scores closer to +1 are considered mesenchymal-like tumors, EMT scores closer to -1 are considered epithelial tumors. In the GSE10245 cohort [5], 35 NSCLC samples were scored as epithelial and 23 as mesenchymal on the hgu133plus2 platform using two probe-sets (209108-at and 209109-s-at).

**TSPAN6 correlation to EMT signature in pancreatic cancer.**

The Collison dataset was processed from normalized gene expression data of the PDA UCSF tumors defined in GSE17891 with clinical survival and censoring information for 27 patients [2]. Similar analysis was conducted on the GSE11838 cohort [1]. Patients with status "alive" were assigned as censored with days to last follow up. We performed a log-rank test using the

survival R package [11]. The minimal p-value was defined based on a log-rank test to split the patient cohorts into a *TSPAN6* low and *TSPAN6* high expression group.

**Statistics.**

Statistical analysis using gene expression data was performed with R version 2.5.1 and BioConductor version 1.8. The remaining statistical analyzes were performed on SPSS 18.0. For the Kaplan Meier survival analysis, a Log rank test was performed. Normally distributed data was statistically analyzed using unpaired two-tailed Student's t-test for single comparisons, and one-way or two-way analysis of variance - ANOVA - for multiple comparisons. ANOVA analyzes were followed by Bonferroni's post hoc tests. Data that was ordinary was analyzed using unpaired two-tailed Mann-Whitney test. The statistical tests undertaken and the p values are indicated in each figure legend. $p \leq 0.05$ was considered to indicate statistical significance. Data are shown as mean values ± standard error of the mean (s.e.m). The numbers of mice per group used in each experiment are annotated in the corresponding figure legends as n.

## Legends to Supplementary Figures

### Supplementary Figure 1: TSPAN6 cooperates with H-RasV12 to induce cell invasion in normal human mammary epithelial cells.

Representative phase constrast microscope images of *SCRIB-shRNA*, *DLG1-shRNA* and *TSPAN6-shRNA* + low or high *H-RASV12* acini in 3D matrigel cultures, showing the formation of invasive acini. Experiments were conducted in triplicate. Note increased invasive spiking (indicated by black arrows) when the knockdown of *SCRIB*, *DLG1* or *TSPAN6* was combined with both low and high levels of *H-RASV12*. Interestingly, some invasive spiking was also noted in the *TSPAN6*-knockdown cells without *H-RASV12* (black arrow). Scale bar = 100μM.

### Supplementary Figure 2. TSPAN6 affects Ras and EGFR activation in pancreatic cancer cells.

**(a)** Top panel: representative confocal microscopy image of mCherry-expressing MIA-PACA-2 cells treated with doxycycline (Dox) (10mg/ml) for 48 hours. Lower panel, FACS sorting of MIA-PACA-2 cells stably expressing TSPAN6-mCherry upon 48h doxycycline (10mg/ml) treatment. **(b)** ELISA Ras activation assay of Dox treated or untreated *TSPAN6*-MIA-PACA-2 serum starved cells left unstimulated or stimulated with EGFα. Experiments were carried out in triplicate. **(c)** Phospho-protein array analysis. *TSPAN6*-MIA-PACA-2 cells were untreated or treated with Dox for an initial 48 hours. The cells were serum starved for an additional 24 hours in the absence or presence of Dox. The cells were then untreated or stimulated with EGFα for 5 minutes. Whole cell lysates were analyzed on a human phospho-kinase antibody array and the blots quantified. **(d)** Phospho-EGFR analysis. *TSPAN6*-PANC1 cells were prepared as in Figure S2A and then stimulated with EGFα for the indicated time periods. Total cell lysates were analyzed by Western blot with antibodies against phospho-EGFR and total EGFR. Bar graphs show quantifications of the Western blot. **(e)** Cell proliferation analysis: *TSPAN6* expressing MIA-PACA-2 or PANC1 stable cells were untreated or treated with Dox for 48 hours. The cells were then serum starved for an additional 12 hours in the absence or presence

of Dox. Subsequently, BrdU was added and the cells stimulated with EGFα for an additional 4 hours. BrdU incorporation was analyzed by ELISA. **(f)** Scratch assays. Representative images of the *in vitro* scratch assay. Non-induced or doxycycline-induced 80-90% confluent monolayers of *TSPAN6*-PANC1 cells were treated with mitomycin C for 2 hours to block proliferation. Images show cells 24 hours after scratching. All experiments were preformed twice.

## Supplementary Figure 3. TSPAN6 overexpression suppresses the expression of EMT markers in pancreatic cancer cells.

mRNA expression of N-cadherin, Vimentin or Slug EMT markers in EGFα-stimulated PANC-1 or MIA-PACA-2 pancreatic cancer cells are suppressed by the expression of *TSPAN6*. **(a)** N-cadherin and Vimentin mRNA expression in PANC-1 cells after stimulation with EGFα, with or without Dox to induce *TSPAN6* expression, and with or without erlotinib (EGFR inhibitor). After 24hrs, cells were harvested and mRNA expression levels of the N-cadherin and Vimentin EMT markers were determined by q-RT-PCR. **(b)** Slug mRNA expression in PANC-1 or MIA-PACA-2 cells 8hrs after stimulation with EGFα with or without Dox to induce *TSPAN6* expression, and with or without erlotinib. Suppression of EMT marker expression by erlotinib indicates that EGFα-EGFR signaling is required for the EMT phenotype. Experiments were preformed twice.

## Supplementary Figure 4. TSPAN6 overexpression suppresses growth and metastasis of human pancreatic cancer cells *in vivo*.

Volumes of pancreatic tumors after orthotopic injection of either un-induced or *TSPAN6* induced **(a)** MIA-PACA-2 or **(d)** PANC-1 cells. Tumor volumes were measured at 10 weeks for MIA-PACA-2 and 12 weeks for PANC-1 after orthotopic injection into the pancreas of *nu/nu* host mice. **(b)** Images and representative H&E stainings of primary pancreatic tumors following orthotopic injection of uninduced (*TSPAN6* OFF) or *TSPAN6*-induced (*TSPAN6* ON) MIA-

PACA-2 cells. **(c)** Representative H&E-stained liver sections of *TSPAN6* ON and *TSPAN6* OFF MIA-PACA-2 pancreatic cancer cells 10 weeks after orthotopic injection. The arrow indicates a metastatic lesion in the liver. Scale bar = 50μM. **(e)** Representative pancreas sections (H&E staining) of mice following orthotopic injection of *TSPAN6* ON and *TSPAN6* OFF PANC1 cells. Scale bar = 200μM. Data are from 12 weeks after orthotopic injection into *nu/nu* hosts. Metastatic lesions are indicated by arrows. Experiments were preformed twice.

## Supplementary Figure 5. Whole body knockout of *Tspan6* accelerates *Kras*$^{V12G}$-driven lung cancer.

**(a)** Targeting strategy for whole body *Tspan6* knockout mice. **(b)** Western blotting showing efficient loss of Tspan6 protein in lung tumors derived from *Tspan6*$^{-/y}$ *Kras*$^{G12D}$ mice. β-actin is shown as a loading control. **(c)** Quantification of overall tumor burden in *Tspan6*$^{-/y}$ *Kras*$^{G12D}$ and *Tspan6*$^{+/y}$ *Kras*$^{G12D}$ littermates. For quantification of overall tumor burden, hyperplasia, adenomas and adenocarcinomas were scored automatically by an algorithm programmed and executed using the Definiens software suite program. Three planes from each lung were stained with H&E and analyzed in a blinded fashion. n=5 for each time point analyzed. Data are shown as means ± s.e.m. *P < 0.05; N.S.= not significant (Student's t-test). **(d)** Representative immunostaining for the proliferation marker Ki67 on lung tumor sections from *Tspan6*$^{-/y}$ *Kras*$^{G12D}$ and *Tspan6*$^{+/y}$ *Kras*$^{G12D}$ littermates, 8 weeks after infection. Scale bar = 100μM. **(e)** Quantification of % Ki67$^{+}$ cells in hyperplastic regions, adenomas, and adenocarcinomas at 4 and 8 weeks after Adeno-Cre (Ad-Cre) infection. Three planes from each lung were immunostained with anti-Ki67 and analyzed in a blinded fashion. Data are shown as means ± s.e.m. * P < 0.05; ** P < 0.01; N.S.= not significant (Student's t-test). Experiments were preformed twice.

## Supplementary Figure 6. Conditional gene targeting of *Tspan6*.

**(a)** Conditional gene targeting strategy of *Tspan6. Tspan6* exons 2-6 are flanked by *LoxP* sites (black triangles) and Cre-mediated deletion results in a frameshift of the reading frame giving

rise to a truncated protein. The Neo-cassette is a selection marker for positive electroporation of the targeting construct into ES cells and is flanked by FRT sites (black circles). The Neo-cassette was then removed using Flpe-FRT mediated excision. DTA, diphtheria toxin selection gene. Of note, both mouse and human *Tspan6* genes are located on the X chromosome. **(b)** Southern blot verification of the correctly targeted *Tspan6*$^{flox}$ allele in electroporated ES cells. DNA was cut with *EcoRV* to obtain the wild type allele (16356bp) or the *Tspan6flox allele* (9633bp). **(c)** Schematic representation of genetic alterations taking place *in vivo* upon inhalation of Ad-Cre viral supernatants **(d)** Western blotting showing loss of Tspan6 protein in lung tumors derived from *Tspan6*$^{fl/y}$ *Kras*$^{G12D}$ mice. β-actin is shown as a loading control. Experiments were preformed twice.

## Supplementary Figure 7. *Tspan6* deletion does not affect angiogenesis, immune activation or apoptosis.

Representative *in situ* immunostaining for CD3ε, FoxP3, cleaved Caspase 3, and CD31 expression in lung tumors from *Tspan6*$^{fl/y}$ *Kras*$^{G12D}$ (n=5) and *Tspan6*$^{+/y}$ *Kras*$^{G12D}$ mice (n=5). Right panels show quantification of the respective immunomarkers in lung tumors at 4 and 8 weeks after Ad-Cre inhalation. Three planes from each lung were stained and analyzed in a blinded fashion. Data are shown as means ± s.e.m. N.S.= not significant (Student's t-test). Foxp3 and CD31 images, scale bars = 100μM; caspase 3 (CP3) and CD3ε images, scale bars = 200μM. Experiments were preformed twice.

Supplementary references:

1 Balagurunathan Y, Morse DL, Hostetter G, Shanmugam V, Stafford P, Shack S *et al*. Gene expression profiling-based identification of cell-surface targets for developing multimeric ligands in pancreatic cancer. Mol Cancer Ther 2008; 7: 3071-3080.

2 Collisson EA, Sadanandam A, Olson P, Gibb WJ, Truitt M, Gu S *et al*. Subtypes of pancreatic ductal adenocarcinoma and their differing responses to therapy. Nature medicine 2011; 17: 500-503.

3 Debnath J, Muthuswamy SK, Brugge JS. Morphogenesis and oncogenesis of MCF-10A mammary epithelial acini grown in three-dimensional basement membrane cultures. Methods 2003; 30: 256-268.

4 Dow LE, Elsum IA, King CL, Kinross KM, Richardson HE, Humbert PO. Loss of human Scribble cooperates with H-Ras to promote cell invasion through deregulation of MAPK signalling. Oncogene 2008; 27: 5988-6001.

5 Kuner R, Muley T, Meister M, Ruschhaupt M, Buness A, Xu EC *et al*. Global gene expression analysis reveals specific patterns of cell junctions in non-small cell lung cancer subtypes. Lung Cancer 2009; 63: 32-38.

6 Oft M, Peli J, Rudaz C, Schwarz H, Beug H, Reichmann E. TGF-beta1 and Ha-Ras collaborate in modulating the phenotypic plasticity and invasiveness of epithelial tumor cells. Genes & development 1996; 10: 2462-2477.

7 Reichmann E, Ball R, Groner B, Friis RR. New mammary epithelial and fibroblastic cell clones in coculture form structures competent to differentiate functionally. The Journal of cell biology 1989; 108: 1127-1138.

8 Schramek D, Sigl V, Penninger JM. RANKL and RANK in sex hormone-induced breast cancer and breast cancer metastasis. Trends Endocrinol Metab 2011; 22: 188-194.

9 Schulte J, Weidig M, Balzer P, Richter P, Franz M, Junker K *et al*. Expression of the E-cadherin repressors Snail, Slug and Zeb1 in urothelial carcinoma of the urinary bladder: relation to stromal fibroblast activation and invasive behaviour of carcinoma cells. Histochem Cell Biol 2012; 138: 847-860.

10 Tan TZ, Miow QH, Miki Y, Noda T, Mori S, Huang RY *et al*. Epithelial-mesenchymal transition spectrum quantification and its efficacy in deciphering survival and drug responses of cancer patients. EMBO Mol Med 2014; 6: 1279-1293.

11 Therneau TM, Grambsch PM. Modeling Survival Data: Extending the Cox Model. Springer-Verlag, New York 2000.

# Supplementary Figures

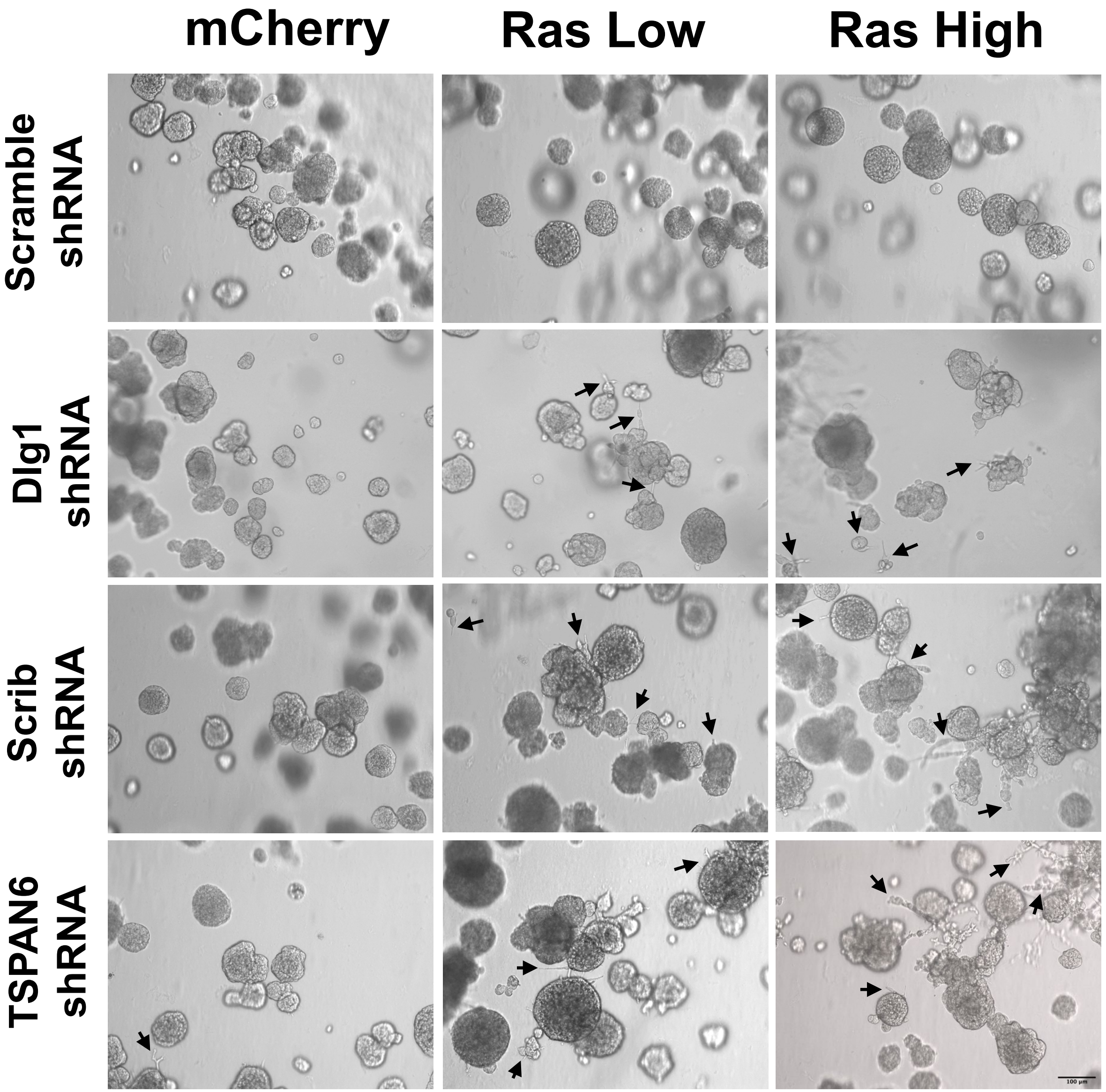


Supplementary Figure 1

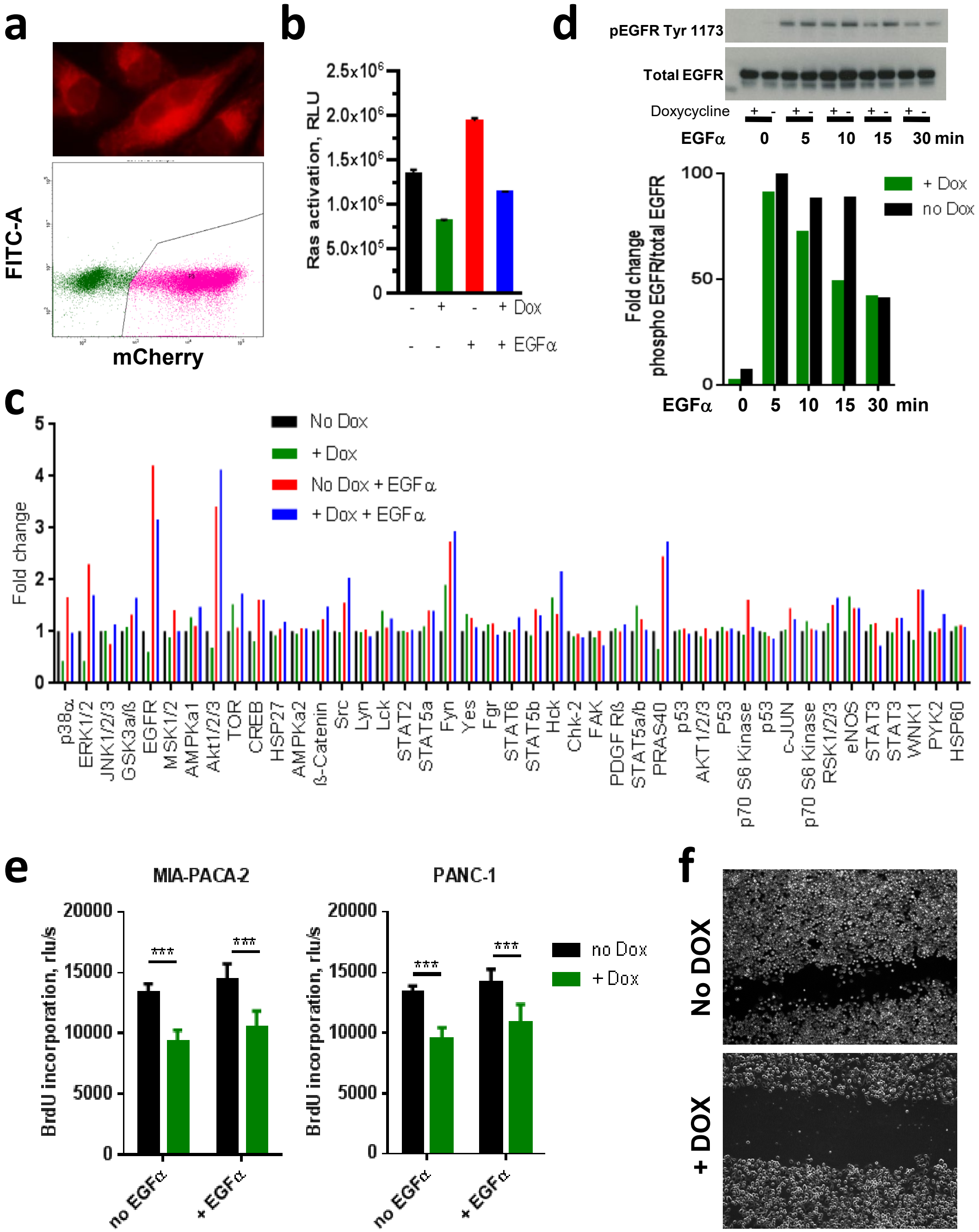


**Supplementary Figure 2**

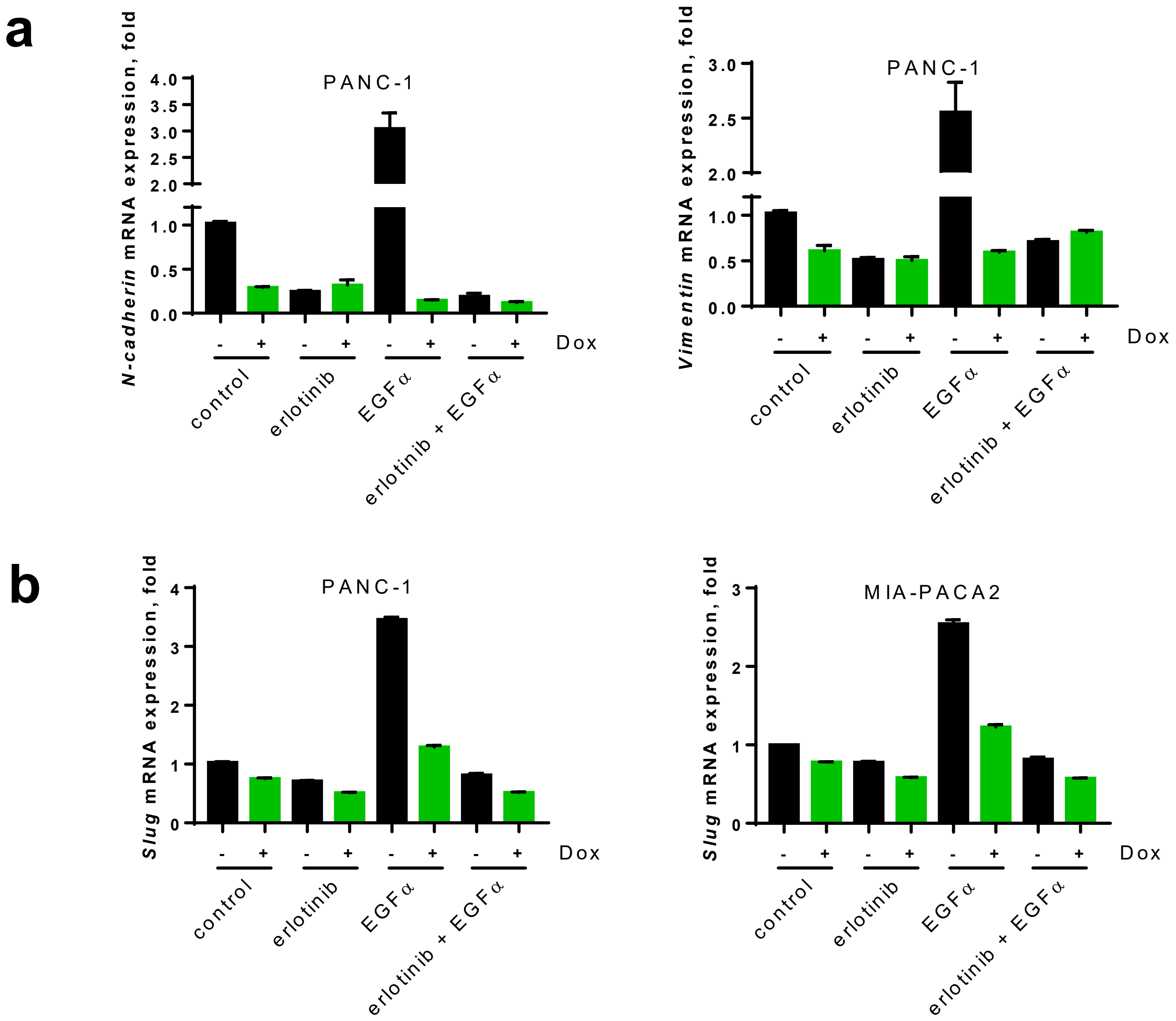


**Supplementary Figure 3**

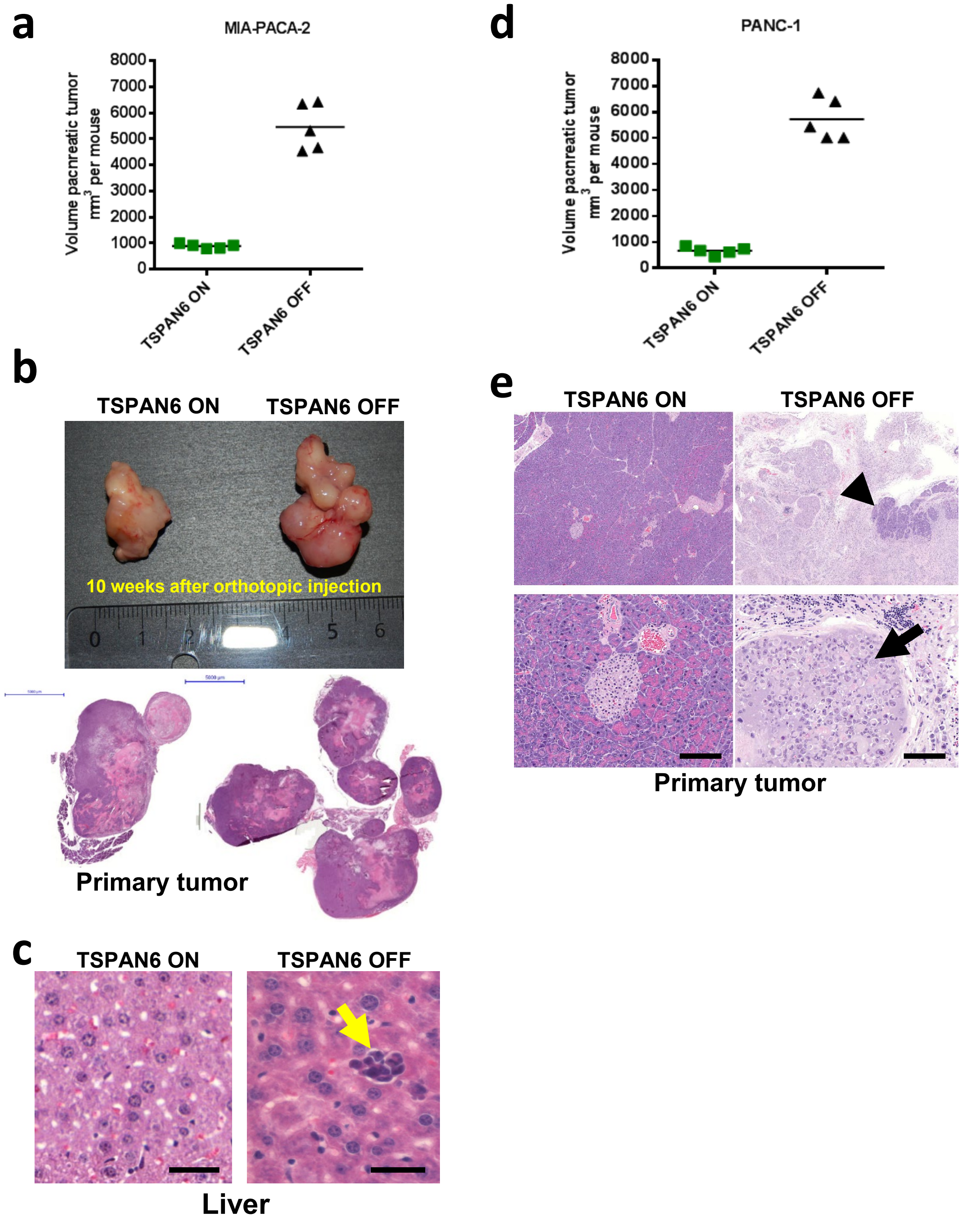


**Supplementary Figure 4**

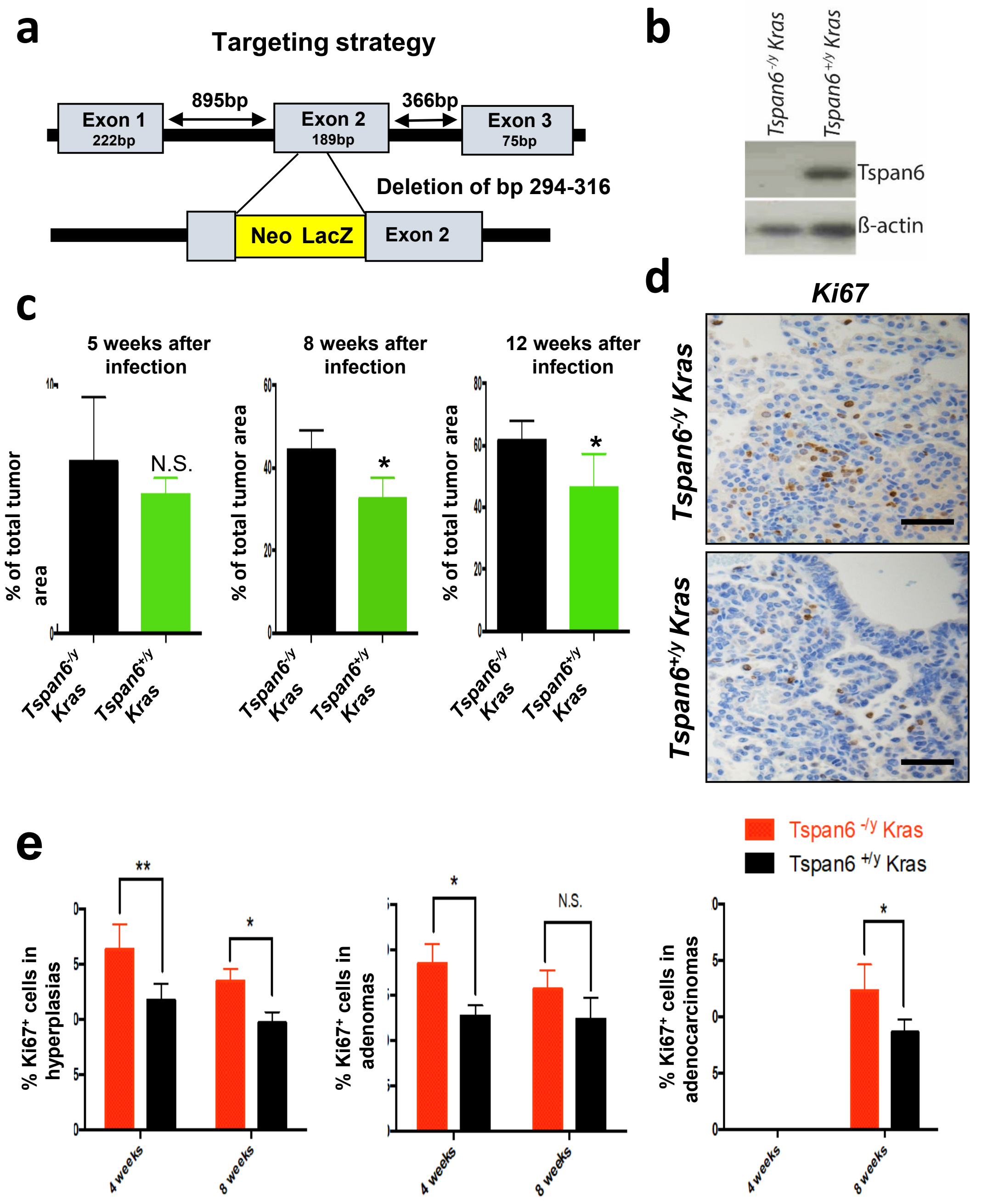


**Supplementary Figure 5**

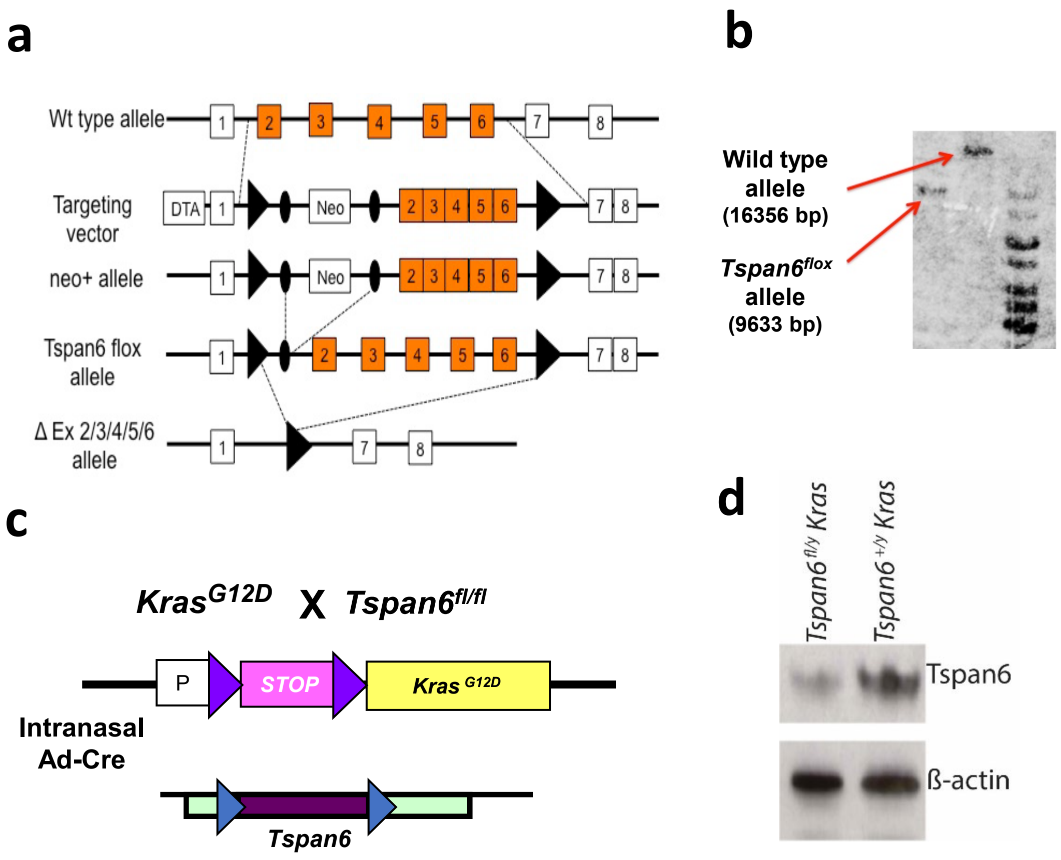


**Supplementary Figure 6**

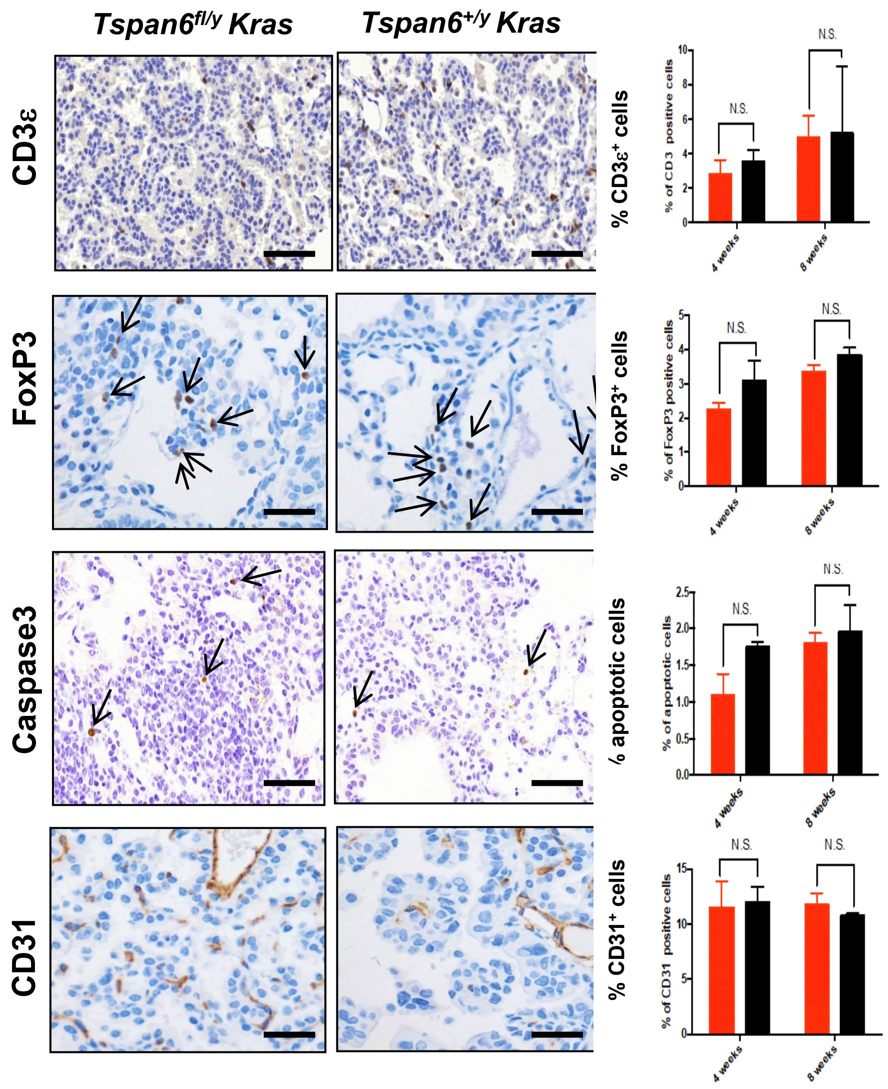


Supplementary Figure 7
